# CD40 Activation Rescues Antiviral CD8^+^ T Cells from PD-1-Mediated Exhaustion

**DOI:** 10.1371/journal.ppat.1003490

**Published:** 2013-07-11

**Authors:** Masanori Isogawa, Josan Chung, Yasuhiro Murata, Kazuhiro Kakimi, Francis V. Chisari

**Affiliations:** Department of Immunology and Microbial Science, The Scripps Research Institute, La Jolla, California, United States of America; Nationwide Children's Hospital, United States of America

## Abstract

The intrahepatic immune environment is normally biased towards tolerance. Nonetheless, effective antiviral immune responses can be induced against hepatotropic pathogens. To examine the immunological basis of this paradox we studied the ability of hepatocellularly expressed hepatitis B virus (HBV) to activate immunologically naïve HBV-specific CD8^+^ T cell receptor (TCR) transgenic T cells after adoptive transfer to HBV transgenic mice. Intrahepatic priming triggered vigorous *in situ* T cell proliferation but failed to induce interferon gamma production or cytolytic effector function. In contrast, the same T cells differentiated into cytolytic effector T cells in HBV transgenic mice if Programmed Death 1 (PD-1) expression was genetically ablated, suggesting that intrahepatic antigen presentation *per se* triggers negative regulatory signals that prevent the functional differentiation of naïve CD8^+^ T cells. Surprisingly, coadministration of an agonistic anti-CD40 antibody (αCD40) inhibited PD-1 induction and restored T cell effector function, thereby inhibiting viral gene expression and causing a necroinflammatory liver disease. Importantly, the depletion of myeloid dendritic cells (mDCs) strongly diminished the αCD40 mediated functional differentiation of HBV-specific CD8^+^ T cells, suggesting that activation of mDCs was responsible for the functional differentiation of HBV-specific CD8^+^ T cells in αCD40 treated animals. These results demonstrate that antigen-specific, PD-1-mediated CD8^+^ T cell exhaustion can be rescued by CD40-mediated mDC-activation.

## Author Summary

Hepatitis B virus (HBV) infection is responsible for more than 500,000 deaths annually as a result of the immune-mediated chronic liver damage it induces. The HBV specific CD8^+^ T cell response contributes to the pathogenesis of liver disease and viral clearance, and the failure to induce and/or sustain a vigorous CD8^+^ T cell response results in viral persistence and causes chronic necroinflammatory liver disease. To understand how the HBV-specific CD8^+^ T cell response is generated in response to intrahepatically expressed HBV, we generated T cell receptor transgenic mice whose CD8^+^ T cells are specific for HBV core or HBV envelope antigens. We find that these T cells are primed in the liver when they are adoptively transferred into HBV transgenic mouse recipients whose livers produce infectious virus particles, and that they proliferate vigorously in situ but do not differentiate into functional effector T cells after antigen recognition. Functional differentiation is suppressed by dominant negative regulatory signals, including PD-1, unless they are suppressed by anti-CD40 activation of myeloid dendritic cells.

## Introduction

Rapid clonal expansion of CD8^+^ T cells in response to antigenic challenge is a hallmark of adaptive immunity and a crucial element of host defense. Activation and differentiation of T cells are largely determined by their initial encounter with antigen-presenting cells (APCs), and the resultant responses range from full activation and memory T cell differentiation to clonal exhaustion or deletion, depending on the nature and abundance of inductive signals that T cells decode from APCs during priming [1], [2]. These events generally occur in secondary lymphoid organs because naïve T cells are usually not primed in nonlymphoid tissues [2]. The liver is, however, an exception to this rule, due to the unique architecture of the hepatic sinusoid which is characterized by a discontinuous endothelium, the absence of a basement membrane, and a very slow flow rate [3]–[5], allowing circulating T cells to make prolonged direct contact with resident liver cells including hepatocytes [6]. Furthermore, the liver is replete with diverse and unique antigen presenting cell populations, including liver sinusoidal endothelial cells (LSECs) [7], [8], hepatic stellate cells (HSCs) [9], Kupffer cells [10], [11], conventional and plasmacytoid dendritic cells [12]–[14], all of which are capable of priming and/or tolerizing naïve T cells, at least in vitro. Thus, because of its unique immunological environment, antigens expressed and/or processed in the liver appear to be more accessible to T cells than those in other nonlymphoid organs [4], [15].

The hepatitis B virus (HBV) is a noncytopathic, enveloped, double-stranded DNA virus that causes acute and chronic hepatitis and hepatocellular carcinoma [16], [17]. Similar to other noncytopathic viruses, the clearance of HBV requires functional virus-specific CD8^+^ T cell responses [18]. Using the HBV transgenic mouse [19] as a model to study the impact of intrahepatic antigen recognition by HBV-specific CD8^+^ T cells, we have shown that adoptively transferred HBV-specific memory CD8^+^ T cells rapidly secrete IFNγ upon antigen recognition in the liver, thereby inhibiting HBV replication [20]. Subsequently, PD-1 is upregulated in the intrahepatic CD8^+^ T cells and they stop producing IFNγ, start expressing granzyme B (GrB) and undergo massive expansion [21] thereby mediating a necroinflammatory liver disease and terminating viral gene expression whereupon the intrahepatic CD8^+^ T cell population contracts, liver disease abates and IFNγ production returns [21].

While the foregoing studies illustrate the profound impact of intrahepatic antigen recognition on the distribution, expansion and effector functions of memory CD8^+^ T cells, they do not address the response of immunologically naïve CD8^+^ T cells to antigen recognition in the liver. Indeed, the literature reveals significant differences between naïve and memory CD8^+^ T cells in terms of the peptide:MHC complex concentration and costimulation required for activation and the development of their proliferative and cytokine secretion potentials, cytolytic activity and their migratory range [2], [22]. While T cell priming to viruses that do not infect conventional pAPCs is believed to occur in lymphoid organs via cross-priming [1], [2], [23], [24], the consequences of naïve T cell priming by hepatocellularly expressed viral antigen are less well understood.

In the current study, we used transgenic mice whose CD8^+^ T cells express T cell receptors (TCRs) specific for the HBV nucleocapsid (COR) and envelope (ENV) proteins to study the early intrahepatic immunological events that are likely to occur during HBV infection. By analyzing the response of naïve COR- and ENV-specific TCR transgenic CD8^+^ T cells to hepatocellularly presented HBV antigens in vivo after adoptive transfer into HBV transgenic mice whose hepatocytes produce all the HBV gene products and secrete infectious HBV virions [19], and in vitro after cocultivation with primary HBV transgenic mouse hepatocytes, we show that HBV-specific naïve CD8^+^ T cells are primed in the liver by HBV^+^ hepatocytes and proliferate vigorously in situ, but do not differentiate into functional effector T cells unless PD-1 signaling is genetically ablated. Importantly, when the same T cells are transferred into HBV transgenic mice whose myeloid dendritic cells (mDCs) were simultaneously activated by agonistic antibodies against CD40 (αCD40), PD-1 induction is suppressed and the T cells differentiate normally, inhibit HBV antigen expression, and cause liver disease. Collectively, these results indicate that CD40-mediated activation of mDCs can rescue the effector functions of PD-1-inhibited naïve CD8^+^ T cells, apparently by suppressing the negative regulatory signals that are triggered by antigen recognition in the liver. These results imply that the balance achieved between these two opposing forces may regulate the pathogenesis and outcome of HBV and other hepatotropic virus infections.

## Results

### Generation of HBV COR93-specific and ENV28-specific TCR transgenic mice

**Figure 1:**
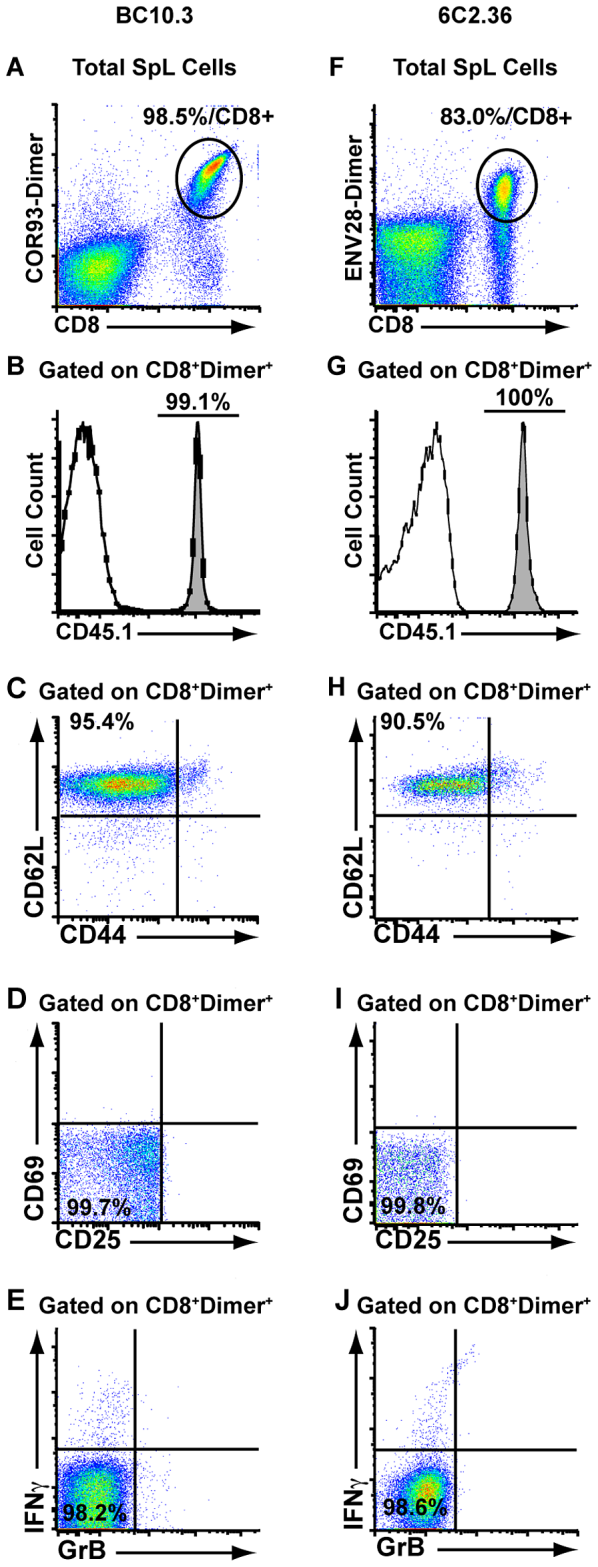
Expression and phenotype of HBV-specific CD8^+^ T cells in T cell receptor (TCR) transgenic mice. Spleen cells were isolated from TCR transgenic mouse lineages BC10.3 and 6C2.36 and the frequencies of K^b^-restricted COR93-specific and L^d^-restricted ENV28-specific CD8^+^ T cells were analyzed using peptide-pulsed K^b^-, and L^d^-dimers respectively (Figure 1A and Figure 1F). Splenic COR93- and ENV28-specific CD8^+^ T cells from respective TCR transgenic mice were gated by CD8^+^ and COR93-dimer and ENV28-dimer staining as shown in Figure 1A and 1F, and analyzed for the expression of CD45.1, CD62L, CD44, CD69, and CD25 (Figures 1B–1D and 1G–1I). Spleen cells from BC10.3 and 6C2.36 TCR transgenic mice were stimulated with COR93 and ENV28 peptide for 5 hours in vitro and CD8^+^ T cells were analyzed for the expression of IFNγ and Granzyme B (GrB) by intracellular cytokine staining (1E and 1J).

A K^b^-restricted CD8^+^ CTL clone (BC10) that recognizes an epitope located between residues 93–100 in the HBV core protein (MGLKFRQL) (COR93) was generated from a Balb/c (H-2^d^) by C57BL/6 (H-2^b^) F1 hybrid (CB6F1) mouse that was immunized by standard DNA-prime/vaccinia boost immunization as previously described [21], [25]. Importantly, when in vitro core peptide-activated BC10 T cells (1×10^7^/mouse) were adoptively transferred into HBV transgenic mice (lineage 1.3.32) that express all of the HBV antigens and replicate HBV in the liver and kidney [19], they inhibited HBV replication, and caused liver disease on day 1 after adoptive transfer (data not shown) as previously described after adoptive transfer of polyclonal COR93-specific effector memory CD8^+^ cells [21]. TCRα (Vα13.1JαNEW06) and β (Vβ8.1Jβ1.2) cDNA clones derived from BC10 were inserted into TCR expression cassettes [26], and injected into fertilized CByB6F2 eggs to generate BC10 TCR transgenic mice. Two founders, BC10.1 and BC10.3 carrying both TCRα and β transgenes were derived, and lineage BC10.3 was chosen for further backcrossing based on its superior allelic exclusion rate (data not shown). The BC10.3 TCR transgenic (TCRtg) mice were backcrossed more than 10 times onto C57BL/6 (B6) background, and then mated once with CD45.1 mice (H-2^b^) so that the TCR transgenic T cells could be easily followed by anti-CD45.1 antibody staining. As shown in Figures 1A and 1B, >98% of the splenic CD8^+^ T cells (33.5% of total spleen cells) in these mice were COR93-specific and CD45.1 positive as determined by staining with COR93-multimers and CD45.1 staining. As expected, they were phenotypically characterized as CD44^−^, CD62L^high^, CD25^−^, CD69^−^, (Figures 1C and 1D) and fewer than 2% of them produced IFNγ or expressed Granzyme B (GrB) after 5 hours peptide stimulation in vitro (Figure 1E), indicating that they were in fact naïve T cells.

We also generated a lineage of transgenic mice whose CD8^+^ T cells express TCRs specific for the well-described L^d^-restricted ENV28 epitope [27], [28]. The TCRs of these mice consist of Vα4.1JαNEW and Vβ1.1Jβ2.5 chains cloned from CD8^+^ ENV28-specific CTL clone 6C2, whose functional properties have been extensively characterized [20], [27]–[29]. Lineage 6C2.36 was chosen for further characterization and backcrossed onto the Balb/c background for at least 6 generations and then mated once with CD45.1 mice (H-2^b^). As shown in Figure 1F, approximately 83% of splenic CD8^+^ T cells (20% of total spleen cells) in lineage 6C2.36 are ENV28-specific and all of them were CD45.1 positive (Figure 1G). Again, virtually all the ENV28-specific CD8^+^ T cells were CD44^−^, CD62L^high^, CD25^−^, CD69^−^, (Figures 1H and 1I), and they did not express IFNγ or GrB after peptide stimulation (Figure 1J), indicating that they are naïve T cells.

### Naïve HBV-specific CD8^+^ T cells expand vigorously in the liver but do not differentiate into effector T cells

To examine the response of HBV-specific naïve CD8^+^ T cells to hepatocellularly expressed HBV, we adoptively transferred 3–5×10^6^ COR93-specific naïve CD8^+^ T cells from the spleen of BC10.3 TCR transgenic donor mice into HBV transgenic lineage 1.3.32 recipient mice [19], [20]. Groups of 3–4 mice were sacrificed at various time points after adoptive transfer, and their intrahepatic, lymph nodal, and splenic lymphocytes were analyzed for the total number of COR93-specific CD8^+^ T cells and the extent to which they coexpress Granzyme B (GrB) and IFNγ either directly ex vivo or after in vitro stimulation by cognate COR93 peptide. To determine the functional capabilities of the adoptively transferred COR93-specific CD8^+^ TCR transgenic T cells during a systemic infection in vivo, we also studied their response to cognate HBcAg antigen produced by MHC-matched nontransgenic mice that had been infected 2 hours before adoptive transfer with 2×10^7^ pfu of a recombinant vaccinia virus that expresses the HBV nucleocapsid protein (cVac) [30].

**Figure 2:**
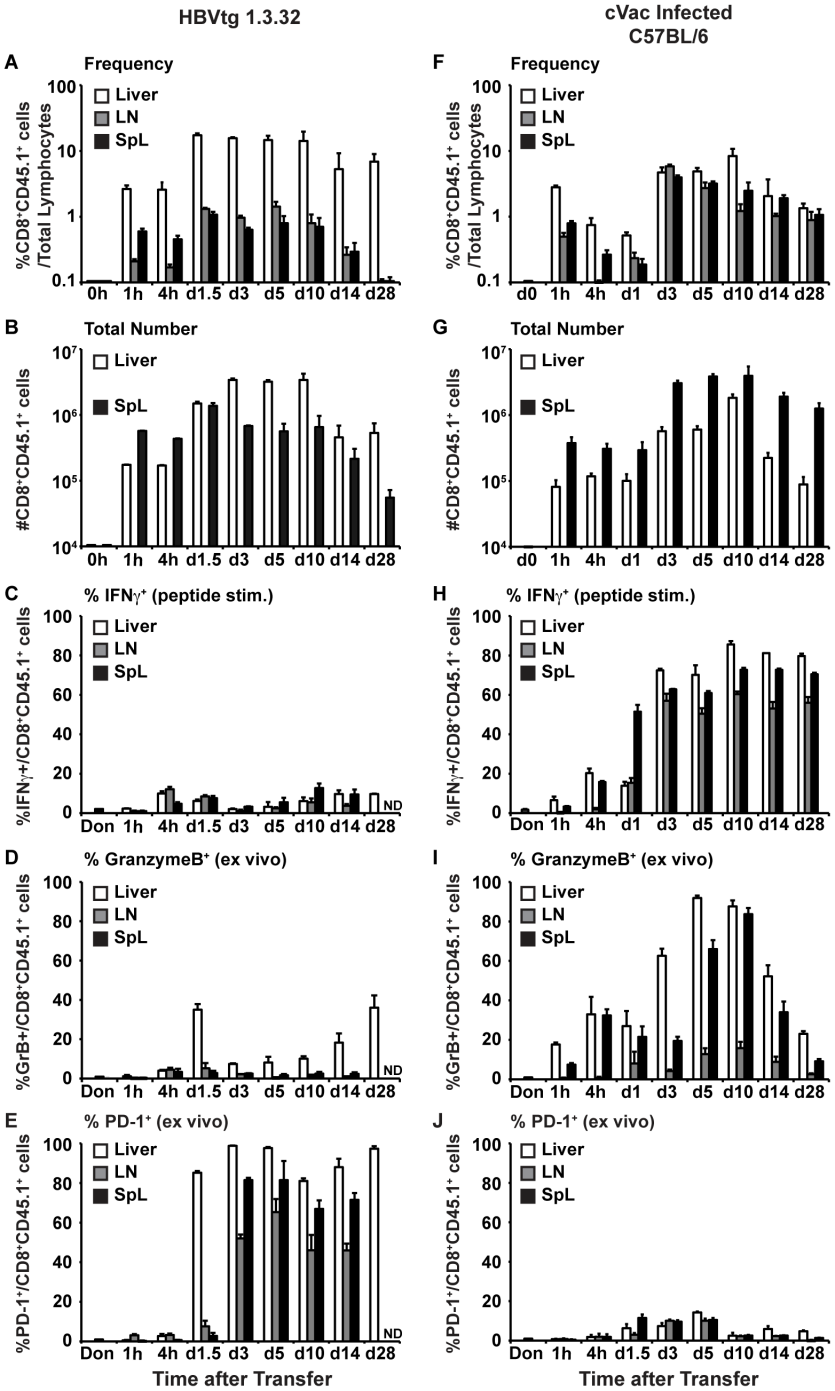
Functional characterization of COR93-specific CD8^+^ T cells. COR93-specific CD8^+^ T cells were analyzed for their ability to expand, produce IFNγ, and express Granzyme B (GrB) in the liver (white), lymph nodes (gray), and spleen (black) at various time points after adoptive transfer to HBV transgenic mice lineage 1.3.32 (left panel) and cVac infected nontransgenic recipients (right panel). Figures (A) and (F) show the frequency COR93-specific CD8^+^ T cells (i.e. CD8^+^CD45.1^+^ cells), while Figure (B) and (G) show the absolute number of COR93-specific CD8^+^ T cells. Figures (C) and (H) show the fraction of in vitro IFNγ -producing COR93-specific CD8^+^ T cells, and Figures (D) and (I) show the fraction of ex vivo Granzyme B expressing CD8^+^ T cells. Figures (E) and (J) show the fraction of PD-1 positive CD8^+^ T cells. The data represent mean ± SD of three mice.

As shown in Figure 2, COR93-specific CD8^+^ T cells were detectable in the liver of HBV transgenic mice as early as 1 hour after adoptive transfer, and they rapidly accumulated in the liver, constituting more than 17% of total intrahepatic lymphocytes on days 1.5 and 3 (Figure 2A white bars) and showing greater than a 20-fold increase in their absolute numbers between the 1 hour and 3 day time points (Figure 2B white bars). After rapid expansion, the number of intrahepatic COR93-specific CD8^+^ T cells remained relatively stable up to day 10, after which they decreased more than 10-fold by day 14 (Figure 2B) but still constituted a large fraction of total intrahepatic CD8^+^ T cells on day 28 (Figure 2A). In contrast, the COR93-specific CD8^+^ T cells in the lymph nodes and spleen expanded much less vigorously between the 1 hour and 3 day time points than their counterparts in the liver, although the absolute number of COR93-specific CD8^+^ T cells was greater in the spleen than the liver at 1 hour and 4 hour time points (Figure 2B). COR93 specific CD8^+^ T cells started disappearing from the lymph nodes and spleen on day 14, and became almost undetectable on day 28 (Figures 2A and 2B; gray and black bars). In contrast, in cVac infected nontransgenic C57BL/6 mice, the frequency of COR93-specific CD8^+^ T cells was similar in the liver, lymph nodes, and spleen (Figure 2F), and fewer COR93-specific CD8^+^ T cells were detectable in the liver than the spleen (Figure 2G) at all time points tested. These results suggest that the COR93-specific CD8^+^ T cells were primed and accumulated preferentially in the antigen expressing liver of HBV transgenic mice rather than peripherally as in the cVac infected nontransgenic mice.

Strikingly, despite vigorous expansion (Figures 2A and 2B), the COR93-specific CD8^+^ T cells in the liver, lymph nodes and spleen of the HBV transgenic mice did not produce IFNγ either directly ex vivo (data not shown) or after 5 hours peptide stimulation (Figure 2C) at any time point examined, and their ability to express GrB was severely compromised as well (Figure 2D). In contrast, the intrahepatic, lymph nodal and splenic COR93-specific CD8^+^ T cells in the cVac infected nontransgenic recipients were able to produce IFNγ in response to 5 hours COR93-peptide stimulation, and expressed GrB directly ex vivo (Figures 2H and 2I). These data suggest that adoptively transferred HBV-specific naïve T cells preferentially expand in the HBV transgenic liver, but the expanding T cells are functionally impaired. Interestingly, virtually all the intrahepatic COR93-specific CD8^+^ T cells in HBV transgenic mice strongly expressed the co-inhibitory molecule PD-1 on day 1.5 ex vivo and remained so until day 28 (Figure 2E), while PD-1 expression was virtually absent in their counterparts in cVac infected nontransgenic animals (Figure 2J), suggesting that PD-1 signaling may have contributed to the functional impairment of intrahepatic COR93-specific CD8^+^ T cell responses in HBV transgenic mice.

**Figure 3:**
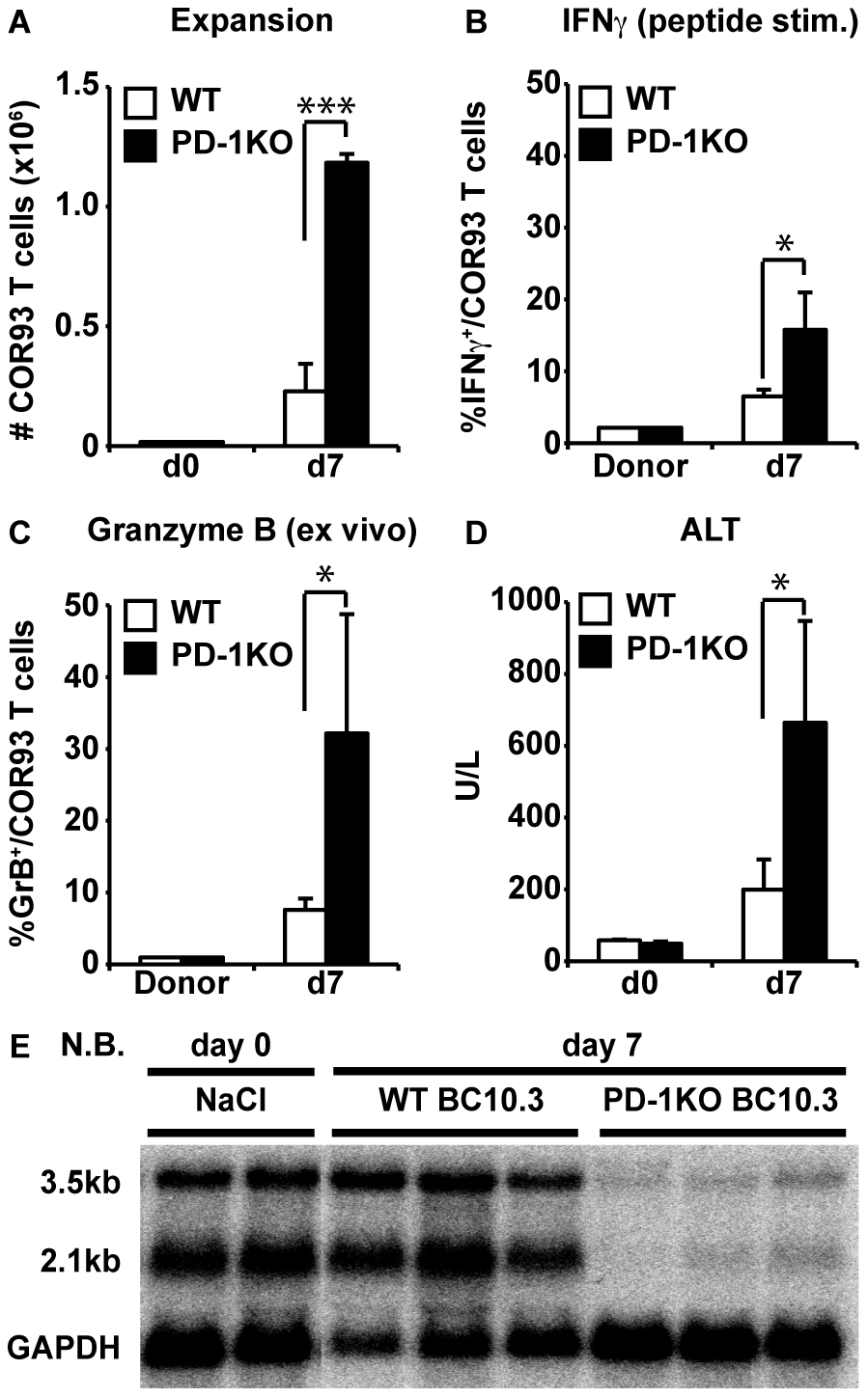
PD-1 deficient COR93-specific CD8^+^ T cells develop cytolytic ability in the liver of HBV transgenic mice. HBV transgenic mice were adoptively transferred with spleen cells from wild type (white) or PD-1 deficient (black) BC10.3 TCR transgenic mice. Mice were sacrificed on day 7 after adoptive transfer to analyze intrahepatic COR93-specific CD8^+^ T cell for the total number (A), the fraction of in vitro IFNγ producing (B), and ex vivo GrB expressing (C) CD8^+^ T cells. Serum alanine aminotransferase (sALT) activity in the same HBV transgenic mice is expressed as units/liter (D). The data represent mean ± SD of three mice. (E) Northern blot analysis of total liver RNA isolated from the same mice. GAPDH was used to normalize the amount of RNA bound to membrane. *P<0.05, **P<0.01, ***P<0.001.

To determine if the dysfunctional T cell responses in the HBV transgenic liver reflect active suppression of functional differentiation by PD-1 signaling, the COR93-specific TCR transgene was crossed for two generations onto a MHC class I matched PD-1 deficient background (kindly provided by Dr. Arlene Sharpe, Harvard Medical School) [31], yielding PD-1 deficient COR93-specific TCR transgenic animals. Equal numbers of PD-1 deficient and wild type COR93-specific naïve CD8^+^ T cells were adoptively transferred into HBV-transgenic mice, and analyzed for expansion, IFNγ producing ability and Granzyme B (GrB) expression on day 7 after adoptive transfer. The results were correlated with the degree of liver damage and HBV gene expression monitored by serum alanine aminotransferase (ALT) activity and HBV gene Northern Blot (NB) analysis, respectively. As shown in Figure 3A, PD-1 deficient COR93-specific CD8^+^ T cells expanded much more vigorously in the liver than wild type COR93-specific CD8^+^ T cells, and a larger fraction of PD-1 deficient T cells expressed IFNγ and Granzyme B (Figure 3B and C) and they induced a more severe liver disease, monitored as serum alanine aminotransferase (ALT) activity (Figure 3D). Furthermore, HBV gene expression was significantly reduced in the recipients of PD-1 deficient COR93-specific CD8^+^ T cells but not in the wild type T cell recipients (Figure 3E), reflecting the superior cytolytic and interferon gamma-producing activity of the PD-1 deficient cells. Collectively, these results indicate that PD-1 signaling suppresses the expansion and functional differentiation of HBV-specific CD8^+^ T cells after antigen recognition in the liver.

### Intrahepatic priming of HBV-Specific naïve T cells after adoptive transfer into HBV transgenic mice

Because the hepatocytes in HBV transgenic mice replicate HBV at high level and release viral particles and subviral antigens into the circulation [19], HBV derived antigen could be presented to naïve T cells either by the hepatocytes themselves or by professional antigen presenting cells (pAPCs) that acquire virus particles and/or subviral antigens in the liver or in peripheral lymphoid organs. Therefore, the expansion of dysfunctional HBV-specific CD8^+^ T cells in the liver could reflect T cell priming and expansion in the liver, or the intrahepatic accumulation of T cells that were previously primed in the lymph nodes. To distinguish between these alternatives, we monitored the expression of activation markers (CD69 and CD25) on HBV-specific CD8^+^ T cells in the liver, lymph nodes and spleen at very early time points (1 hour, 4 hours and day 1) after adoptive transfer into HBV transgenic mice, and the results were compared with the expression of these activation markers on the HBV-specific CD8^+^ T cells in the cVac infected nontransgenic animals.

**Figure 4:**
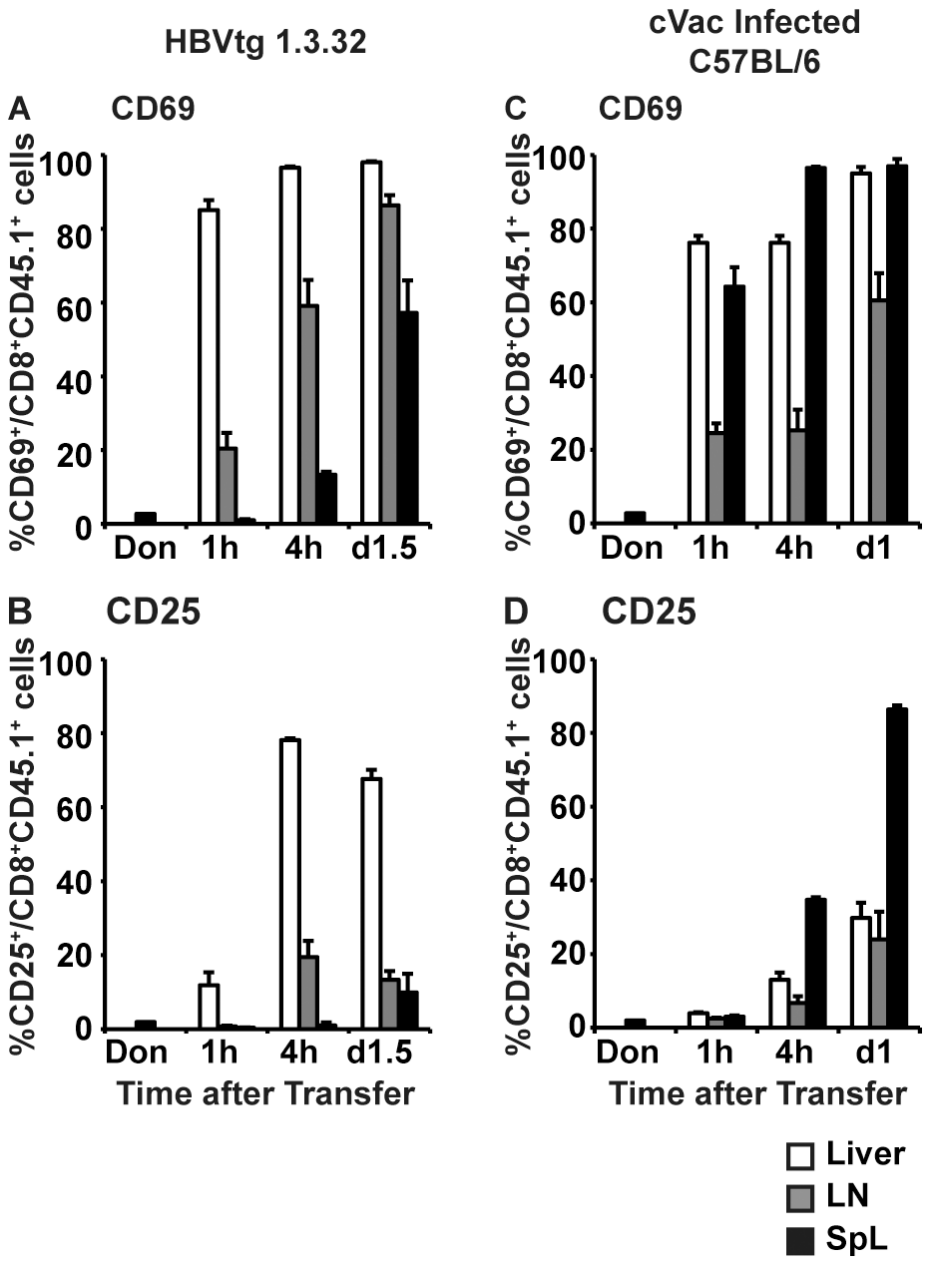
Kinetics of COR93-specific T cell activation. The kinetics of activation marker expression by COR93-specific CD8^+^ T cells in the liver (white), lymph nodes (gray), and spleen (black) were examined at indicated time points after adoptive transfer of 2×10^7^ of spleen cells from BC10.3 TCR transgenic mice into HBV-transgenic mice lineage 1.3.32 (A and B) and nontransgenic mice infected with 2×10^7^ pfu vaccinia virus expressing the HBV core protein (cVac) (C and D). COR93-specific CD8^+^ T cells were identified as CD8^+^CD45^+^ cells and the fraction of CD69 and CD25 expressing COR93-specific CD8^+^ T cells were depicted. The data represent mean ± SD of at least three mice.

As shown in Figure 4A (white bars), within 1 hour after adoptive transfer, approximately 85.0% of the intrahepatic COR93-specific CD8^+^ T cells in the HBV transgenic mice expressed the very early activation marker CD69, suggesting that nearly all the COR93-specific T cells that entered the liver rapidly recognized antigen. By 4 hours, virtually all the intrahepatic COR93-specific CD8^+^ T cells in the transgenic mice were CD69 positive, and a large fraction of them also began to express CD25 (Figure 4B, white bars), the IL-2α receptor that is required for high affinity binding of IL-2 [32], suggesting that they were fully activated and prepared to proliferate. In contrast, CD69 expression by COR93-specific CD8^+^ T cells in the lymph nodes (gray bar) and spleen (black bars) occurred later (Figure 4A) than their intrahepatic counterparts (Figure 4A), and fewer nodal and splenic COR93-specific CD8^+^ T cells expressed CD25 (Figure 4B, gray and black bars), suggesting that naïve HBV-specific CD8^+^ T cell activation primarily occurred in the HBV-expressing liver and that these intrahepatically primed T cells subsequently trafficked to the lymph nodes and spleen. In contrast, COR93-specific CD8^+^ T cells in cVac infected nontransgenic recipients rapidly upregulated CD69 in the spleen and the liver as early as 1 hour after adoptive transfer (Figure 4C). Interestingly, CD25 expression in cVac infected nontransgenic mice was mainly observed on the splenic COR93-specific CD8^+^ T cells (Figure 4D), suggesting that the activation of COR93-specific CD8^+^ T cells during systemic vaccinia infection is largely splenic. None of these changes occurred in uninfected control nontransgenic recipients (data not shown), indicating that they were antigen specific events. Collectively, these results suggest that hepatocellularly expressed HBV antigen primes naïve T cells in the liver.

**Figure 5:**
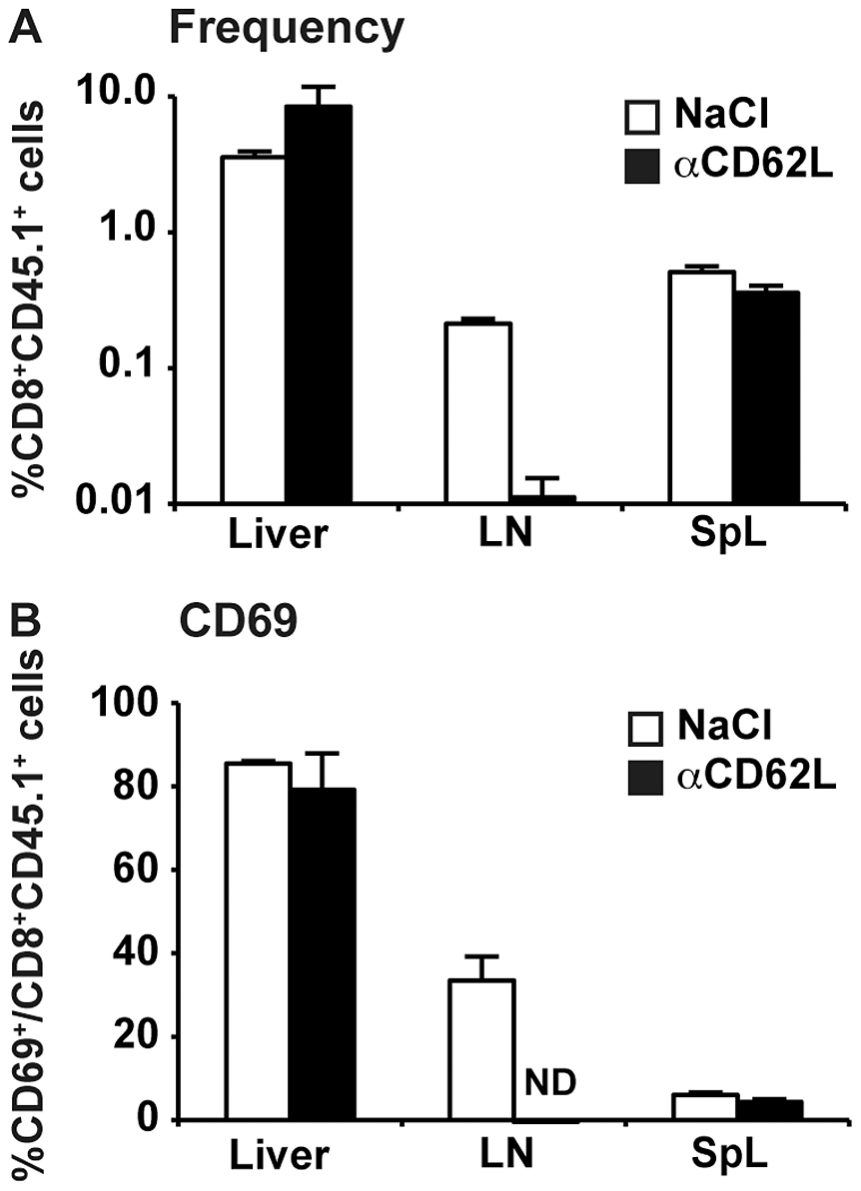
Intrahepatic accumulation and activation of COR93-specific CD8^+^ T cells in HBV transgenic mice is independent of T cell homing to the lymph nodes. Groups of 3–4 lineage 1.3.32 HBV-transgenic mice were treated with anti-CD62L antibodies (αCD62L) at 16 and 4 hours before adoptive transfer of naïve COR93-specific CD8^+^ BC10 T cells. 1 hour later, the mice were sacrificed and organs were harvested to analyze the frequency (A) and CD69 expression (B) of the transferred T cells in the liver, lymph nodes, and spleen. The data represent mean ± SD of three mice.

Next, groups of 4 HBV-transgenic mice received intraperitoneal injections of either saline or anti-CD62L antibodies (αCD62L), that are known to block naïve T cell homing to the lymph nodes [33]–[35], followed by naïve COR93-specific CD8^+^ T cells 16 hours later. The mice were sacrificed 1 hour after adoptive transfer and COR93-specific CD8^+^ T cells were isolated from the liver, lymph nodes, and spleen and analyzed for CD69 expression. As shown in Figure 5A, αCD62L administration completely abrogated the homing of COR93-specific CD8^+^ T cells to lymph nodes, but had no impact on the intrahepatic accumulation of the T cells. Despite the absence of T cell homing to the lymph nodes, COR93-specific CD8^+^ T cells in the αCD62L treated HBV-transgenic mice were fully activated in the liver, similar to those in saline treated recipients (Figure 5B). These results confirm that intrahepatic T cell activation and expansion do not reflect redistribution of T cells that were activated in the lymph nodes.

### Intrahepatic priming of HBV-specific CD8^+^ T cells is primarily mediated by hepatocytes

**Figure 6:**
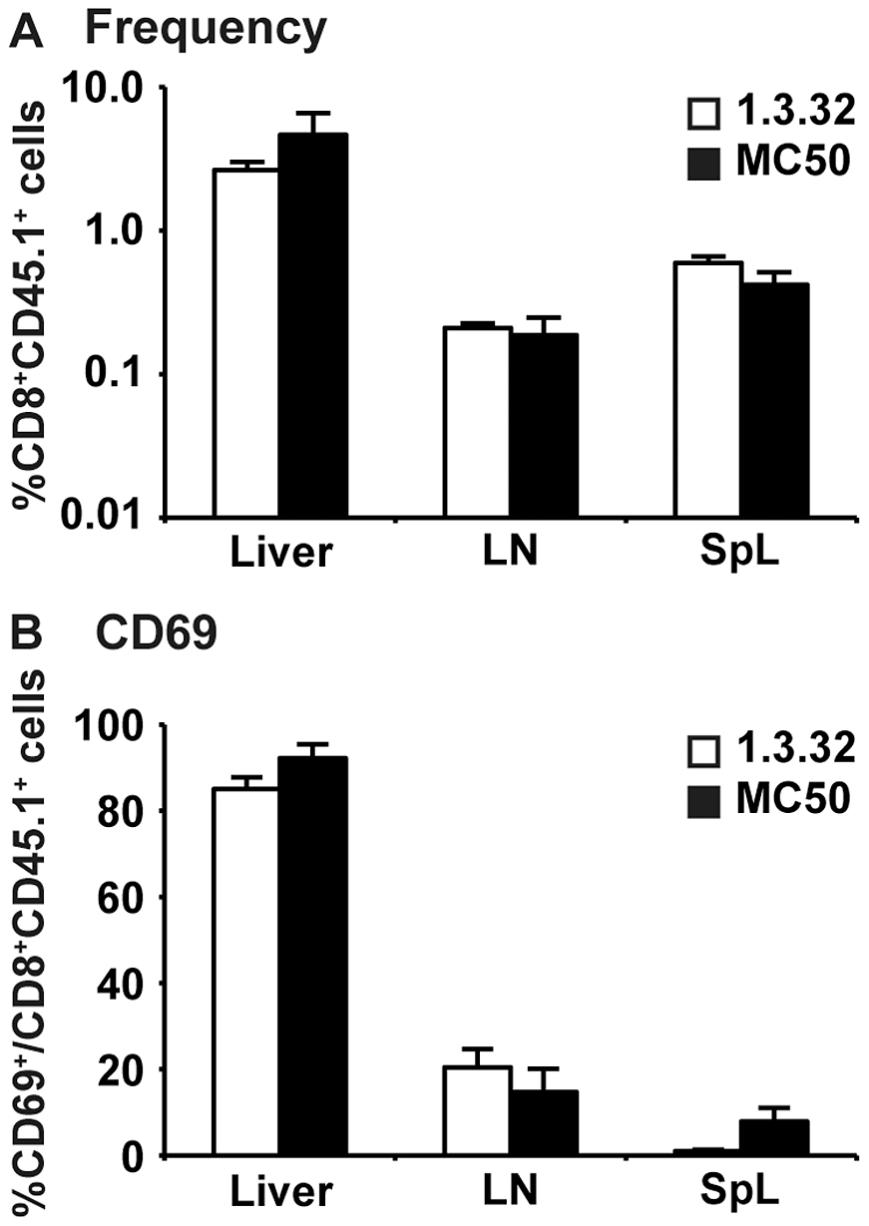
Intrahepatic accumulation and activation of COR93-specific CD8^+^ T cells in HBV transgenic mice. 2×10^7^ of spleen cells were isolated from BC10 TCR transgenic mice, and adoptively transferred into groups of 3–4 lineage 1.3.32 HBV transgenic mice and MUP-core 50 (MC50) transgenic mice. Lineage 1.3.32 replicates HBV and expresses HBcAg in their hepatocytes and secretes virus particles and HBeAg, a secreted viral protein that is highly cross-reactive with HBcAg. Lineage MC50 expresses only HBcAg under the mouse major urinary protein (MUP) promoter, and its expression is restricted to the hepatocytes. 1 hour after adoptive transfer of naïve COR93-specific CD8^+^ BC10 T cells, the mice were sacrificed and organs were harvested to analyze the frequency (A) and CD69 expression (B) of the transferred T cells in the liver, lymph nodes, and spleen. The data represent mean ± SD of three mice.

**Figure 7:**
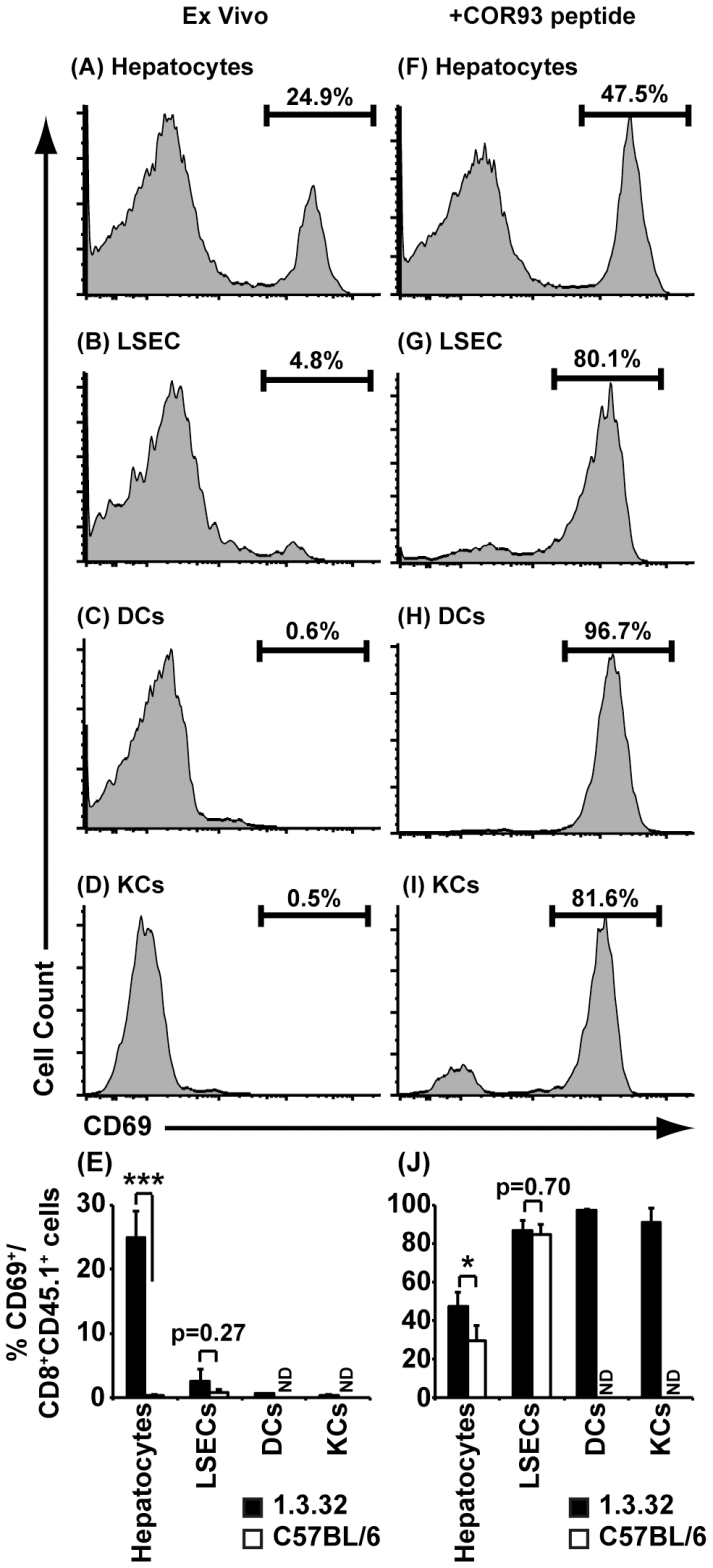
Naïve COR93-specific CD8^+^ T cells are activated by HBV expressing hepatocytes. CD8^+^ T cells were isolated from BC10.3 TCR transgenic mice. Hepatocytes, liver sinusoidal endothelial cells (LSECs), liver dendritic cells (DCs), and Kupffer cells (KCs) were purified from HBV transgenic mice lineage 1.3.32 and nontransgenic littermates as described in the Materials and Methods, and then incubated in culture medium (Ex Vivo; left column) or pulsed with COR93-peptide (+COR93 peptide; right column) for 1 hour. After washing, 2×10^5^ of hepatocytes (A and F) or 1×10^6^ of purified LSECs (B and G), liver DCs (C and H) or Kupffer cells (D and I) from HBV transgenic mice were cocultured with 3×10^5^ of CD8^+^ T cells isolated form BC10.3 TCR transgenic mice. 16 hours later, cells were harvested and CD69 expression on COR93-specific CD8^+^ T cells was monitored by flow cytometric analysis (FACS). A representative histogram illustrating CD69 expression on COR93-specific CD8^+^ T cells after coculture with each cell population from HBV-transgenic mice lineage was shown in panels (A)–(D) and (F)–(I). Experiments were performed three times for each cell population, and bar graphs representing mean ± SD of the three experiments were shown in Figures (E) and (J). Bars represent the percentage of CD69 on COR93-specific CD8^+^ T cells after coculturing with Hepatocytes, LSECs, DCs, and KCs isolated from HBV transgenic mice lineage 1.3.32 (black bars) or with Hepatocytes and LSECs isolated from nontransgenic littermates (white bars). *P<0.05, **P<0.01, ***P<0.001.

Intrahepatic priming of HBV-specific CD8^+^ T cells could reflect recognition of either endogenously synthesized hepatocellular antigen or of antigen that is released by the hepatocytes and internalized, processed and presented by liver sinusoidal endothelial cells (LSEC), Kupffer cells, or dendritic cells that are capable of cross-presentation [8], [10], [12], [36]. In an attempt to identify the antigen presenting cell population responsible for priming COR93-specific CD8^+^ T cells in the liver of HBV transgenic mice, we adoptively transferred COR93-specific naïve T cells into MHC-matched HBV transgenic mice lineages 1.3.32 and MUP-core 50 (MC50) that produce a nonsecretable form of HBcAg, and compared T cell accumulation and activation 1 hour later. Lineage 1.3.32 replicates HBV and expresses HBcAg (which is nonsecretable) in their hepatocytes and it also secretes viral particles and HBeAg, a soluble viral protein that is highly cross-reactive with HBcAg [16], [19]. In contrast, lineage MC50 express only HBcAg whose expression is restricted to hepatocytes [37], reducing the likelihood of antigen presentation by professional antigen presenting cells that acquire secreted viral particles or subviral antigens. As shown in Figure 6, COR93-specific CD8^+^ T cells accumulated similarly in liver, lymph nodes and spleen in both HBV-transgenic mouse lineages (Figure 6A), and the fraction of CD69 positive COR93-specific CD8^+^ T cells in the liver and lymph nodes were comparable in these lineages (Figure 6B). Since HBV core expression in MC50 transgenic mice is restricted to hepatocytes, these results suggest that naïve COR93-specific CD8^+^ T cells were primed by recognition of endogenously synthesized hepatocellular HBcAg. To test this notion, COR93-specific naïve T cells were co-cultured overnight with hepatocytes, LSECs, Kupffer cells, and dendritic cells that were isolated from the liver of HBV transgenic mice lineage 1.3.32 and nontransgenic controls, and then examined for CD69 expression. As shown in Figure 7, approximately 25% of COR93-specific naïve T cells upregulated CD69 when they were cocultured with hepatocytes isolated from HBV transgenic mice (Figure 7A and 7E), whereas fewer than 5% (2.5±1.8%) did so when cocultured with transgenic LSEC, (Figure 7B and 7E) and virtually no T cells expressed CD69 when cocultured with transgenic DCs or KCs (Figure 7C, 7D and 7E) despite the ability of intrahepatic LSECs, DCs and KCs to activate the T cells if the APCs are pulsed with COR93-peptide prior to coculture (Figure 7F–J). As expected, neither hepatocytes nor LSECs isolated from nontransgenic mice stimulated COR93-specific CD8^+^ T cells to express CD69 unless they were first pulsed with COR93-peptide (Figures 7E and 7J, white bars) indicating the antigen specificity of the T cell response to the HBV transgenic hepatocytes. Collectively, these data suggest that intrahepatic priming of COR93-specific CD8^+^ T cells was primarily mediated by endogenously synthesized antigen produced by the HBV-transgenic hepatocytes.

### Intrahepatic priming of functionally defective T cell responses is independent of T cell antigen specificity

**Figure 8:**
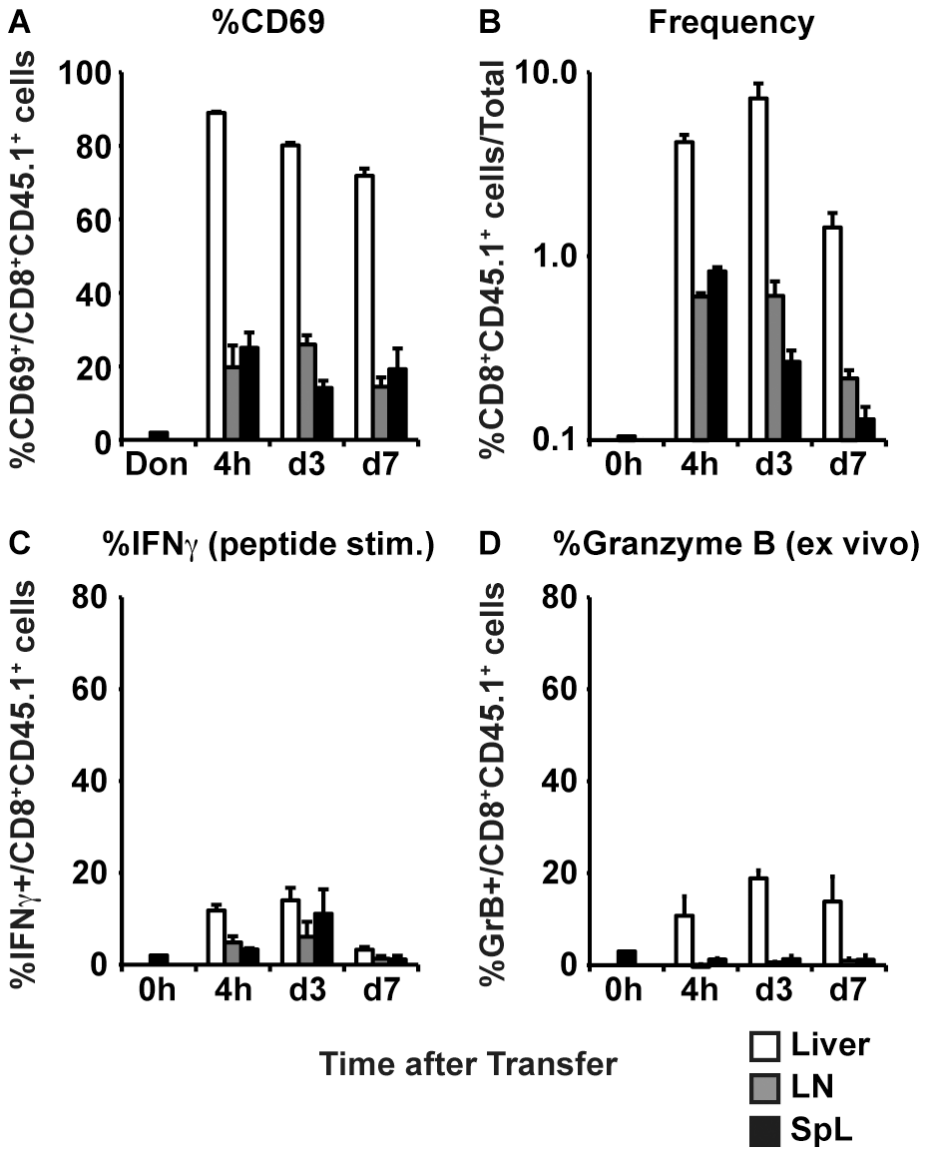
Intrahepatic priming and expansion of functionally defective ENV28-specific CD8^+^ T cells. HBV transgenic mice were adoptively transferred with 5×10^7^ of spleen cells from 6C2.36 TCR transgenic mice, and sacrificed at various time points after adoptive transfer to analyze ENV28-specific CD8^+^ T cells in the liver (white bars), lymph nodes (gray bars) and spleen (black bars) for their activation marker expression (A), expansion (B), and in vitro (i.e. after 5 hours ENV28 peptide stimulation) IFNγ-producing ability (C) and ex vivo Granzyme B expression (D). The data represent mean ± SD of three mice.

To determine if intrahepatic priming of functionally defective CD8^+^ T cells is a general rule or restricted to COR93-specific TCR transgenic T cells, we adoptively transferred naive ENV28-specific T cells from CD45.1-6C2.36 TCRtg mice into MHC-matched HBV transgenic mice and nontransgenic littermates. Groups of 3 mice were sacrificed 4 hours, 3 days and 7 days after adoptive transfer to examine the ENV28-specific CD8^+^ T cell response in the liver (Figure 8; white bars), lymph nodes (Figure 8; gray bars) and spleen (Figure 8, black bars). ENV28-specific naïve CD8^+^ T cells were rapidly activated in the liver of the HBV transgenic mouse recipients (but not in nontransgenic recipients – not shown) as early as 4 hours after adoptive transfer (Figure 8A), suggesting that, like COR93-specific naïve CD8^+^ T cells, adoptively transferred ENV28-specific CD8^+^ naïve T cells are primed in the liver. The intrahepatically primed ENV28-specific CD8^+^ T cells expanded in the liver (Figure 8B), but did not express IFNγ or GrB (Figure 8C and 8D). These results recapitulate the immunological events observed after adoptive transfer of COR93-specific naïve T cells into HBV transgenic mice illustrated in Figures 2 and 4, indicating that intrahepatic T cell priming and the expansion of functionally defective T cells occur irrespective of the antigen specificity or MHC restriction of the T cells. Thus, T cell hyporesponsiveness represents a general outcome induced by intrahepatic T cell priming to endogenously synthesized hepatocellular antigen.

### CD40-Activation of myeloid dendritic cells induces functional differentiation of intrahepatically primed HBV-specific CD8^+^ T cells

Ample evidence suggests that the induction of functional CD8^+^ T cell responses requires the activation of professional antigen presenting cells (pAPCs), which in turn provide secondary signals to naïve T cells [1], [2], [38]. Since the results shown in Figure 7 indicate that HBV-specific naïve T cells were primed by hepatocytes that are not known to express co-stimulatory molecules [39], it is possible that the dysfunctional HBV-specific T cell responses in the HBV transgenic liver reflected the absence of a second signal.

**Figure 9:**
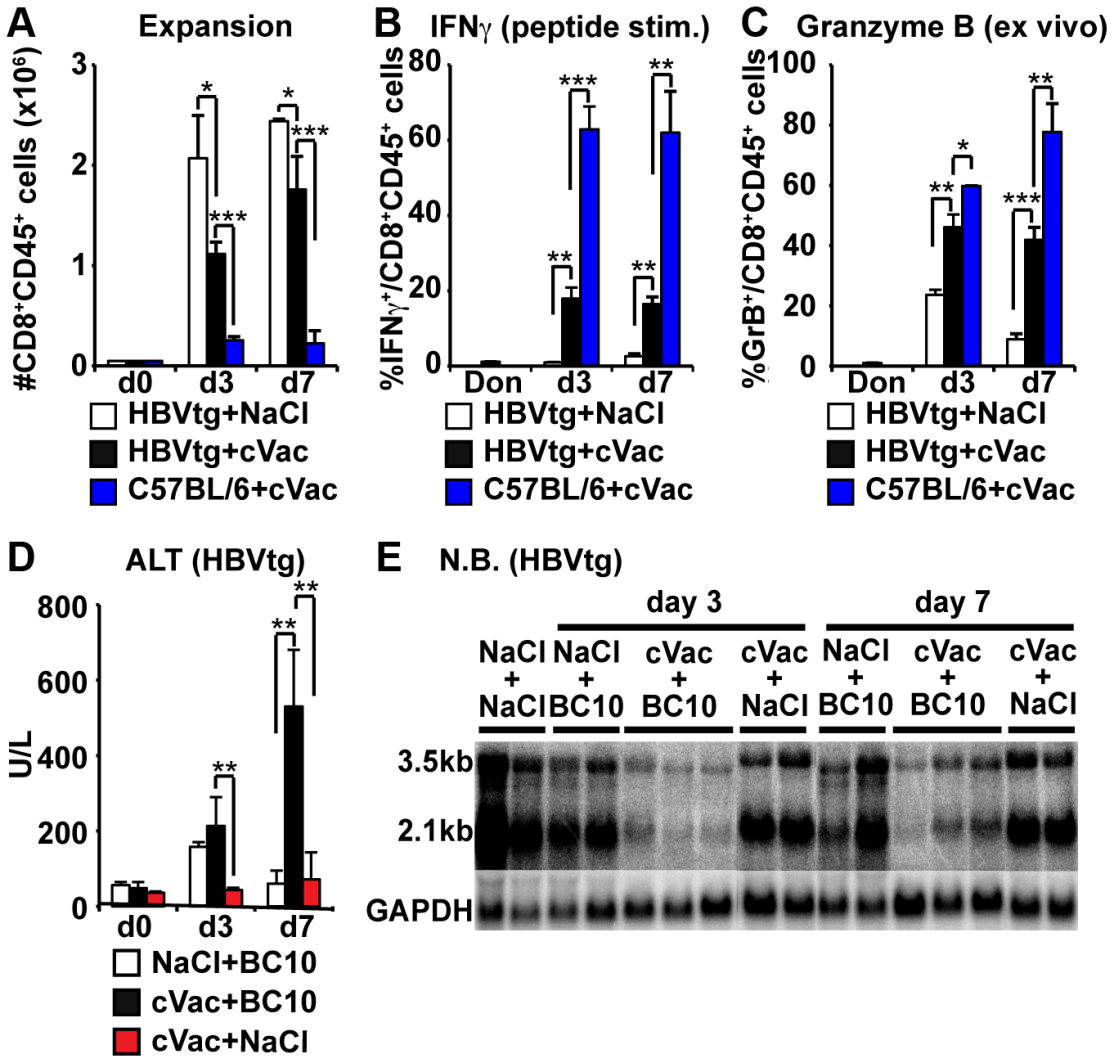
Infection with recombinant vaccinia viruses induces functional differentiation of COR93-specific CD8^+^ T cells in HBV transgenic mice. Groups of 3–4 HBV transgenic mice were treated with either saline (NaCl) or 2×10^7^ pfu of recombinant vaccinia viruses expressing the HBV core antigen (cVac), and one hour later, received 3–5×10^6^ of COR93-specific naïve CD8^+^ T cells. Groups of 3–4 nontransgenic mice were also infected with 2×10^7^ pfu of cVac and then received 3–5×10^6^ of COR93-specific naïve CD8 T cells. In addition, groups of 3–4 HBV transgenic mice were infected with the same titer of cVac without receiving COR93-specific naïve CD8^+^ T cells. On days 3 and 7, transferred COR93-specific CD8^+^ T cells were analyzed for expansion (A), IFNγ production (B) and Granzyme B expression (C) in the liver of HBV transgenic mice treated with saline (white bars), HBV transgenic mice infected with cVac (black bars) and nontransgenic mice infected with cVac (blue bars). The T cell responses in HBV transgenic mice were correlated with the degree of liver disease monitored by serum ALT activity (D) and HBV gene expression in the liver monitored by Northern Blot analysis (E). sALT activity and HBV gene expression in HBV transgenic mice that were infected with cVac without receiving COR93-specific naïve T cells were also shown in (D; red bar) and (E). The data represent mean ± SD of three to four mice (n = 3–4). *P<0.05, **P<0.01, ***P<0.001.

To determine if the differentiation defect of intrahepatically primed HBV-specific CD8^+^ T cells can be rescued by products of the immune response to an exogenous pathogen, COR93-specific naïve T cells were adoptively transferred into HBV transgenic mice that were either treated with saline (NaCl) or infected with 2×10^7^ of cVac 2 hours before transfer, and the results were compared with their differentiation after transfer into nontransgenic recipients that had been infected with 2×10^7^ of cVac 2 hours before transfer. Three and seven days later, mice were sacrificed, and intrahepatic COR93-specific CD8^+^ T cells were analyzed for expansion, IFNγ producing ability and Granzyme B (GrB) expression. The results were correlated with the degree of liver damage and HBV gene expression monitored by serum alanine aminotransferase (ALT) activity and Northern Blot (NB) analysis, respectively. To monitor the impact of cVac infection per se on liver disease and HBV gene expression, HBV transgenic mice were infected with 2×10^7^ of cVac without receiving COR-93-specific naïve CD8^+^ T cells, and they were sacrificed 3 and 7 days later. As expected, COR93-specific naïve T cells expanded vigorously in the HBV transgenic mouse liver but did not express IFNγ or Granzyme B (Figures 9A–9C; white bars). In contrast, cVac infection of HBV transgenic mice triggered IFNγ (Figure 9B; black bar) and Granzyme B (Figure 9C; black bar) expression by a small but significant fraction of the transferred intrahepatic COR93-specific CD8^+^ T cells without significantly increasing their expansion in the liver (Figure 9A; black bars). Note, however, that the frequency of IFNγ^+^ and Granzyme B^+^ CD8^+^ T cells was lower in cVac infected HBV transgenic mice (Figures 9B and 9C, black bars) than in cVac infected nontransgenic recipients (Figures 9B and 9C, blue bars), suggesting that their effector functions were suppressed by continuous hepatocellular antigen recognition, similar to the response we have shown to occur when HBV-specific memory CD8^+^ T cells recognize antigen in the HBV transgenic mouse liver [21].

The COR93-specific CD8^+^ T cells induced only a modest elevation of serum ALT activity in saline injected HBV transgenic mice (Figure 9D), and they had little or no effect on HBV gene expression (Figure 9E) in the liver. In contrast, the CD8^+^ T cell-mediated liver disease was more severe (Figure 9D) and intrahepatic HBV gene expression was strongly suppressed in cVac infected HBV transgenic mice compared to saline treated HBV transgenic controls (Figure 9E). As expected, cVac infection per se did not induce liver disease (Figure 9D,; red bar), nor did it suppress HBV gene expression (Figure 9E), suggesting that the induction of liver disease and the suppression of HBV gene expression in cVac infected HBV transgenic mice after COR93-specific CD8^+^ T cell adoptive transfer were mediated by the T cells. These results suggest that functional differentiation of HBV-specific CD8^+^ T cells in the HBV transgenic mouse liver is sufficiently restored in the context of a systemic virus infection to both cause hepatitis and inhibit viral gene expression.

**Figure 10:**
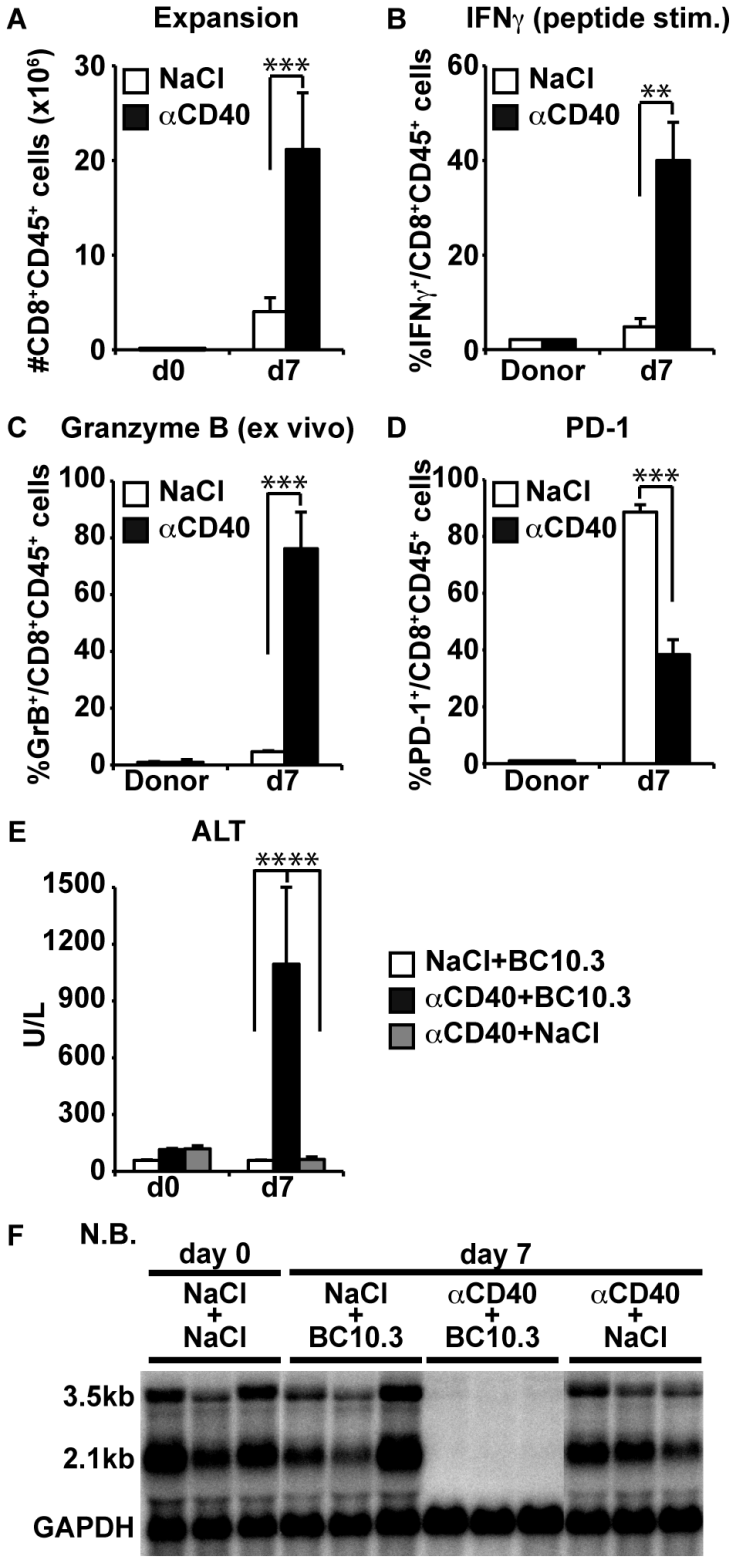
Induction of functional COR93-specific CD8^+^ T cell responses in HBV transgenic mice by CD40 activation. (A)–(D) HBV transgenic mice were treated with either saline (white) or agonistic anti-CD40 antibodies (αCD40) (black), and 16 hours later, 2×10^7^ of spleen cells harvested from BC10.3 TCR transgenic mice (containing approximately 3–5×10^6^ of COR93-specific naive T cells) were adoptively transferred into the HBV transgenic recipients. Mice were sacrificed on day 7 after adoptive transfer to analyze intrahepatic COR93-specific CD8^+^ T cell for the total number (A), the fraction of in vitro IFNγ producing (B), ex vivo GrB expressing (C), and PD-1 expressing (D) CD8^+^ T cells. The data represent mean ± SD of three mice. *P<0.05, **P<0.01, ***P<0.001. (E) Serum alanine aminotransferase (sALT) activity in the HBV transgenic mice described in (A)–(D) is expressed as units/liter. sALT activity in HBV transgenic mice that were treated with αCD40, but did not receive BC10.3 TCR transgenic spleen cells was also shown in gray bars. The data represent mean ± SD of three mice. *P<0.05, **P<0.01, ***P<0.001. (F) Effect of COR93-specific CD8^+^ T cell responses on HBV gene expression in the liver. Northern blot analysis of 20 µg of total liver RNA isolated from the same mice. GAPDH was used to normalize the amount of RNA bound to membrane.

Activation of professional antigen presenting (pAPC) cells is believed to be essential for the induction of functional CD8^+^ T cell responses after virus infections, and several studies have demonstrated that ligation of CD40 induces pAPC activation, resulting in the induction of CD8^+^ T cell responses [40]–[42]. To examine whether CD40 activation could induce functional differentiation of HBV-specific CD8^+^ T cells in this model, we adoptively transferred naïve COR93-specfic CD8^+^ T cells into HBV transgenic mice and nontransgenic controls that had been injected intravenously either with 100 µg/mouse of an agonistic anti-CD40 antibody (αCD40) [43], [44] or with saline (NaCl) 16 hours before transfer. Seven days later, the mice were sacrificed, and intrahepatic COR93-specific CD8^+^ T cells were analyzed for expansion, IFNγ producing ability and Granzyme B (GrB) expression. The results were correlated with the degree of liver damage and HBV gene expression monitored by serum ALT activity and Northern Blot analysis, respectively. As shown in Figure 10A, by day 7, αCD40 treatment increased the intrahepatic expansion of COR93-specific CD8^+^ T cells in HBV transgenic mice by 5 fold compared to the saline treated transgenic animals. Furthermore, by day 7, approximately 40% of intrahepatic COR93-specific CD8^+^ T cells in the αCD40 treated animals produced IFNγ in response to 5 hours in vitro peptide stimulation (Figure 10B), and almost all the COR93-specific CD8^+^ T cells expressed GrB directly ex vivo (Figure 10C), contrasting strikingly to their counterparts in the saline treated animals. Interestingly the induction of T cell effector functions coincided with PD-1 downregulation in intrahepatic COR93-specific CD8^+^ T cells (Figure 10D), suggesting that activation of CD40 signaling counteracted the PD-1 mediated negative signaling. In contrast to these observations, neither the expansion nor the functional differentiation of the COR93-specific CD8^+^ T cells were enhanced in αCD40 treated nontransgenic recipients (not shown). Importantly, αCD40 treated HBV transgenic recipients displayed higher serum ALT activity (Figure 10E) and very strong suppression of intrahepatic HBV gene expression (Figure 10F), after adoptive transfer of naïve COR93-specific CD8^+^ T cells compared to saline treated animals. The induction of severe liver disease and the suppression of HBV gene expression by αCD40 treatment reflect the vigorous expansion and functional differentiation of adoptively transferred COR93-specific CD8^+^ T cells, since these changes were not observed in αCD40 treated transgenic recipients that did not receive naïve COR93-specific CD8^+^ T cells (Figure 10E; gray bars, and Figure 10F). These results suggest that CD40 activation during intrahepatic T cell priming converts T cell hyporesponsiveness into immunity.

**Figure 11:**
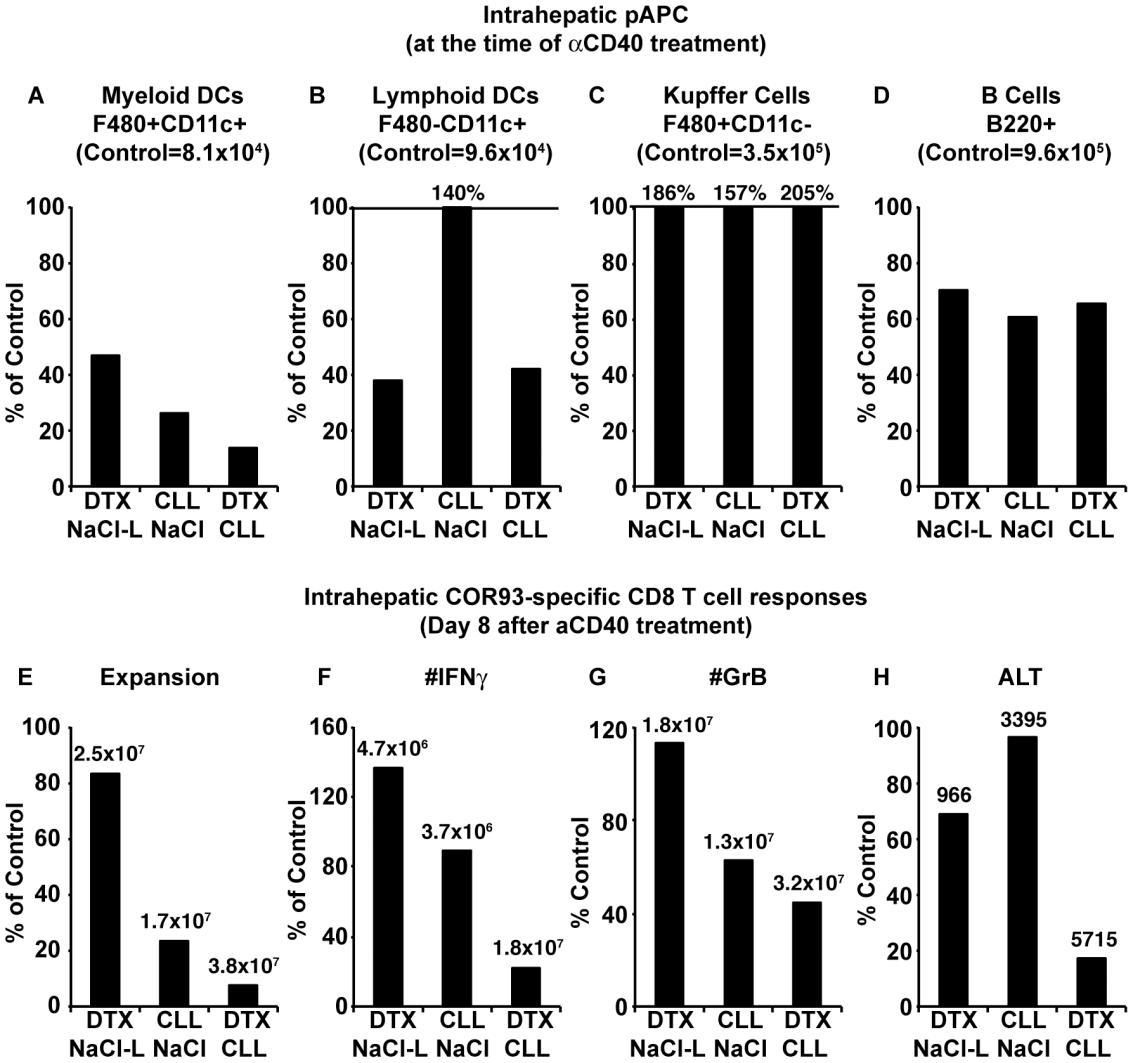
Depletion of myeloid dendritic cells (mDCs) diminishes CD40 activation induced functional differentiation of HBV-specific CD8^+^ T cells. (A)–(D): Four groups of 3 CD11c-DOG mice were treated with saline (NaCl)+control liposome (NaCl-L), Diphtherial Toxin (DTX)+NaCl-L, NaCl+clodronate liposome (CLL) and DTX+CLL, and then sacrificed for the analysis of intrahepatic professional antigen presenting cell (pAPC) population. DTX or saline (NaCl) was administered 1 and 3 days before sacrifice, while CLL or NaCl-L was administered 2 days before sacrifice. The number of myeloid dendritic cells (F480^+^CD11c^+^), lymphoid dendritic cells (F480^−^CD11c^+^), Kupffer cells (F480^+^CD11c^−^), and B cells (B220^+^) were measured by FACS analysis. The percent of control group (NaCl+NaCl-L) in each group (n = 3) was expressed as a bar, and the absolute number of each cell population in the control group (n = 3) was also depicted. (E)–(H); Groups of 3–4 CD11c-DOG HBV transgenic mice were treated with DTX, CLL or DTX+CLL, and then received agonistic anti-CD40 antibody (αCD40). CD11c-DOG HBV-transgenic mice that were treated with NaCl, NaCl-L or NaCl+NaCl-L served as controls for DTX, CLL, or DTX+CLL treated mice, respectively. DTX or NaCl was administered every other day beginning from 3 days before αCD40 administration. CLL or NaCl-L was administered once 2 days before αCD40 administration. On day 1 after αCD40 administration, 2–3×10^6^ of purified COR93-specific naïve CD8^+^ T cells were adoptively transferred into each group. The mice were sacrificed on day 7 after adoptive transfer of COR93-specific naïve CD8^+^ T cells, and the absolute numbers of intrahepatic COR93-specific CD8^+^ T cells, IFNγ-producing CD8^+^ T cells, and Granzyme B positive CD8^+^ T cells, and ALT activity in DTX, CLL, and DTX+CLL treated mice (n = 3–4 for each group) were divided by the corresponding numbers in their respective control group (n = 3–4). Numbers above each bar represent the control values for each condition.

We then attempted to determine the role of professional antigen presenting cells (pAPCs) in αCD40 induced functional differentiation of HBV-specific CD8^+^ T cells. To do so, HBV-transgenic mice were crossed with CD11c.DOG mice that express the human diphtheria toxin (DTX) receptor on CD11c^+^ cells and thus allow depletion of dendritic cells after DTX administration with no signs of toxicity [45]. Groups of three CD11c.DOG-HBV transgenic mice were treated with DTX or saline (NaCl) every other day in combination with single administration of clodronate liposome (CLL) that is known to induce apoptosis of macrophages and DCs in vivo and in vitro [46], [47], or control liposomes (NaCl-L), yielding 4 different groups of mice (i.e. NaCl+NaCl-L, DTX+NaCl-L, CLL+NaCl, and DTX+CLL.) On day 2 after CLL or NaCl-L treatment, we analyzed the numbers of myeloid dendritic cells (mDCs; F480^+^CD11c^+^), lymphoid dendritic cells (lymDCs: F480^−^CD11c^+^), Kupffer cells (F480^+^CD11c^−^) and B cells (B220^+^) in the liver to determine the efficacy of pAPCs depletion. As shown in Figures 11A and 11B, DTX and CLL independently depleted mDCs in the liver and their effects were additive. (Figure 11A), while intrahepatic lymDCs were depleted only by DTX treatment (Figure 11B). Surprisingly, the number of Kupffer cells paradoxically increased when mice were treated with DTX or CLL alone and with both together (Figure 11C). This might reflect that dendritic cell death stimulated proliferation and/or migration of Kupffer cells. None of these treatments significantly reduced the number of intrahepatic B cells (Figure 11D). To examine the impact of pAPC-depletion on αCD40 induced functional differentiation of HBV-specific CD8^+^ T cells, CD11c.DOG-HBV transgenic mice that were pre-treated with DTX, CLL or DTX plus CLL, were injected with αCD40, and 1 day later, adoptively transferred with COR93-specific naïve T cells. The mice were sacrificed on day 7 after adoptive transfer, and the intrahepatic COR93-specific CD8^+^ T cells were analyzed for expansion, IFNγ producing ability and Granzyme B (GrB) expression. The T cell responses were correlated with the degree of liver damage monitored by serum ALT activity. As shown in Figures 11E to 11G, expansion, IFNγ producing ability, and GrB expression of COR93-specific CD8^+^ T cells in αCD40 treated CD11c.DOG transgenic mice were directly correlated with the number of intrahepatic mDCs at the time of αCD40 administration, but not those of intrahepatic lymDCs, Kupffer cells, or B cells, suggesting that mDCs are required for αCD40 induced functional differentiation of HBV-specific CD8^+^ T cells. Taken together, these results suggest that activation of mDCs through the CD40 pathway can overcome PD-1-mediated suppression and induce functional CD8^+^ T cell responses in response to intrahepatically expressed HBV.

## Discussion

The current study examines the impact of hepatocellular antigen presentation on the expansion and functional differentiation of antigen-specific CD8^+^ T cells. Our results revealed that intrahepatic antigen presentation primes functionally defective antigen-specific CD8^+^ T cell responses, that the dysfunctional CD8^+^ T cell response reflects active suppression of expansion and functional differentiation by PD-1 signaling, and that such suppression can be overridden by activating myeloid dendritic cells (mDCs) through CD40 stimulation.

COR93-specific naïve CD8^+^ T cells were rapidly activated in the liver after adoptive transfer into HBV transgenic mice as indicated by more rapid expression of activation markers CD69 and CD25 by intrahepatic COR93-specific CD8^+^ T cells than their lymph nodal and splenic counterparts (Figures 4A and 4B). Hepatocellular activation of the COR93-specific CD8^+^ T cells was not due to hepatic migration of cells that had been activated in lymphoid organs, because the naïve T cells were equally activated when T cell homing to lymph nodes was prevented by anti-CD62L antibody (αCD62L) treatment (Figure 5) and when HBcAg secretion was precluded (Figure 6). Rather, it reflected T cell priming in the liver by recognition of endogenously synthesized hepatocellular antigen (Figure 7). As a consequence of hepatocellular antigen presentation, the COR93-specific CD8^+^ T cells upregulated CD69 and CD25 expression (Figure 4A and B) and expanded vigorously (Figures 2A and 2B) but they were functionally impaired as they did not express IFNγ or Granzyme B (GrB) either directly ex vivo or after 5 hours in vitro peptide stimulation (Figures 2C and 2D). Importantly, hepatocellular T cell priming and the expansion of functionally defective T cells also occurred when ENV28-specific naïve T cells were transferred into HBV-transgenic mice (Figure 8), indicating that hepatocellular T cell priming induces functionally defective T cells responses irrespective of antigen specificity and MHC restriction and illustrating the generality of these observations. The dysfunctional HBV-specific T cell responses likely reflected the suppression of functional differentiation by PD-1 signaling (Figure 3), but such suppression could be overcome by vaccinia virus infection (Figure 9) or simultaneous activation of mDCs via the CD40 signaling pathway (Figures 10 and 11).

While various hepatic cell populations have been shown to contribute to T cell priming in the liver [8], [10], [12], [36], [39], our data suggest that HBV antigen-positive hepatocytes are responsible for priming of HBV-specific CD8^+^ T cells in our system. The fenestrated liver sinusoidal endothelium permits circulating T cells to make direct contact with underlying hepatocytes [6]. Indeed, when hepatitis B envelope (ENV) specific effector CD8^+^ T cells were adoptively transferred into hepatitis B virus (HBV) transgenic mice that express the ENV protein in their hepatocytes, renal tubular epithelium, and choroid plexus cells [48], the ENV-specific effector CD8^+^ T cells were selectively sequestered and specifically activated in the liver where they caused a necroinflammatory disease [4], [29] but not in the other tissues. Importantly, whereas they failed to recognize antigen in the kidney or the CNS when injected intravenously, the CTLs were highly cytopathic for ENV-positive renal tubules and choroid plexus epithelial cells when they were injected directly into those tissues [4]. Furthermore, a series of studies by Bertolino and colleagues suggest that hepatocytes can prime alloantigen specific naïve T cells [15], [35], [39], suggesting that antigen expressed by hepatocytes is highly accessible to circulating native T cells. In those studies, however, the intrahepatic priming of alloantigen specific T cells by hepatocytes was shown to induce rapid T cell deletion [15], [35], [39], contrasting strikingly to the vigorous expansion of HBV-specific CD8^+^ T cell responses described in this study. The basis for the difference is unclear, but it could reflect the different TCR affinity of transgenic T cells and the level of cognate antigen expression in the liver. Nonetheless, in Bertolino's hands and ours, intrahepatic antigen recognition fails to trigger CD8^+^ T cell functional differentiation.

Strikingly, the intrahepatically activated HBV-specific CD8^+^ T cells in the HBV transgenic mice did not secrete IFNγ or display cytotoxic activity (Figures 2C and 2D). Consequently, they did not inhibit HBV replication (data not shown) or gene expression (Figure 3E) or cause a necroinflammatory liver disease (Figure 3D). The lack of effector functions was not due to intrinsic defects of HBV-specific TCR transgenic CD8^+^ T cells or the large number of adoptively transferred T cells, since the same transgenic CD8^+^ T cells differentiated into fully functional effector T cells in nontransgenic recipients that were infected with recombinant vaccinia viruses expressing the HBV core antigen (Figures 2F–2I). Instead, our data suggest that the dysfunctional intrahepatic CD8^+^ T cell response reflects the impact of antigen-induced, PD-1-mediated negative signaling. The intrahepatic HBV-specific CD8^+^ T cells upregulated PD-1 (Figure 2E) in HBV transgenic mice but not in nontransgenic mice infected with cVac (Figure 2J). Furthermore, PD-1 deficient COR93-specific naïve T cells expanded more vigorously than their PD-1 positive wild-type counterparts, and they differentiated into cytotoxic effector T cells in situ, caused severe liver damage, and inhibited HBV gene expression in the liver (Figure 3). These results suggest that HBV-specific CD8^+^ T cells can be primed in the liver by recognition of antigen expressed by hepatocytes but activation of their effector functions is suppressed by PD-1 signaling, consistent with the previous studies reported by us [21], [49] and others [50], [51]. Whether other negative signaling molecules such as CTLA-4 [52], Tim3 [53]–[55], 2B4 [56], [57], IL-10 [58], [59] and TGFβ [60], [61] that are known to suppress antiviral CD8^+^ T cell responses also contributed to the suppression of functional differentiation after intrahepatic priming remains to be determined. In chronic HBV patients, the inhibitory molecule 2B4 and Tim-3 are highly co-expressed with PD-1 on HBV-specific CD8^+^ T cells [62], [63]. Similarly, CTLA-4 is highly expressed on HBV-specific CD8^+^ T cells that express high levels of pro-apoptotic molecule Bim [64]. Furthermore, in vitro blockade of CTLA-4 or Tim-3 signaling appears to restore effector functions of HBV-specific CD8^+^ T cells after in vitro peptide stimulation, and this effect was even enhanced when combined with PD-1 blockade, suggesting that HBV-specific CD8^+^ T cell responses in chronically infected patients are suppressed by several non-redundant mechanisms [62], [64]. Importantly, our data suggest that such suppressive mechanism(s) can be overcome by myeloid dendritic cell (mDC) activation. HBV-specific CD8^+^ T cells differentiated into fully functional effector T cells in recipient HBV transgenic mice that were treated with agonistic anti-CD40 antibodies (αCD40), resulting in liver disease and the inhibition of HBV gene expression (Figure 10). Importantly, mDCs are required for αCD40 induced functional differentiation of HBV-specific CD8^+^ T cells (Figure 11). Collectively, these results suggest that activation of mDCs via CD40 signaling was essential to rescue HBV-specific CD8^+^ T cells from functional suppression through PD-1 and perhaps other regulatory molecules.

Several studies with αCD40 established a model postulating that the αCD40 activates pAPCs that then provide secondary signals to naïve CD8^+^ T cells upon antigen presentation [38], [40], [42]. According to this model, HBV-specific CD8^+^ T cells were programmed to differentiate into functional effector T cells during cross-priming by αCD40-activated pAPCs that acquired circulating HBV particles, subviral antigens or HBV expressing hepatocytes or hepatocyte fragments. However, our preliminary data suggest that hepatic DCs isolated from αCD40 treated HBV-transgenic mice cannot stimulate COR93-specific native T cells to express CD69 (data not shown), suggesting inefficient cross-priming by αCD40-activated pAPCs. Therefore, it is possible that αCD40-activated pAPCs released cytokines such as IL-12 and type I interferons that provide a third signal required for T cell functional differentiation [1], [65], [66]. In line with this notion, Maini and colleagues have recently showed that IL-12 potently augments the capacity of HBV-specific CD8^+^ T cells to produce IFNγ upon in vitro stimulation by cognate antigen in association with down-modulation of PD-1 [67]. Additional studies are required to test these hypotheses.

It remains to be determined if similar events occur during natural HBV infection. Ample evidence suggests that HBV-specific CD8^+^ T cell responses are functionally impaired during chronic HBV infections [51], [68], [69] and the functional impairment is associated with PD-1 expression [68], [70] by HBV-specific CD8^+^ T cells, similar to the transgenic T cells described in this study. Therefore, the expansion of functionally defective CD8^+^ T cells by hepatocellular priming may explain the weak CD8^+^ T cell responses observed during chronic HBV infections. In contrast, approximately 95% of adult onset acute HBV infection is characterized by a vigorous HBV-specific CD8^+^ T cell response. While our data indicate that activation of mDCs through the CD40 pathway can induce functional HBV-specific CD8^+^ T cell responses, it is currently unknown whether, and if so, how the mDCs are activated during natural HBV infection. While CD40 ligand (CD40L) is expressed on a variety of cells including platelets, mast cells, basophils, NK cells, and B cells, antigen-specific CD4^+^ T cells are primarily responsible for activating CD40 expressing cells, particularly professional antigen presenting cells (pAPCs), and CD4 T cell mediated CD40 activation appears essential for cross-priming functional CD8^+^ T cell responses [40]–[42]. Indeed, early priming of HBV-specific CD4^+^ T cells before or during viral spread in HBV-infected chimpanzees appears to be necessary to initiate a functionally efficient CD8^+^ T cell response, and the depletion of CD4^+^ T cells before HBV infection precluded functional T cell priming and caused persistent infection in experimentally infected chimpanzees [70]. Experiments are currently underway to determine whether fully functional CD8^+^ T cell responses can be induced in this transgenic mouse model by providing HBV-specific T cell help.

In summary, the data described herein demonstrate that endogenously synthesized hepatocellular antigen primes functionally defective HBV-specific CD8^+^ T cells via an instructional process involving PD-1 signaling that actively suppresses expansion and functional differentiation of hepatocellularly primed T cells. Importantly, such suppressive mechanisms can be overcome by activating mDCs through the CD40 pathway. Collectively, these results suggest that HBV specific CD8^+^ T cell responses are regulated by the balance between PD-1 mediated inhibitory signaling and stimulatory signals by activated DCs. More experiments are required to determine whether DC activation and/or PD-1 blockade may, individually or together, have therapeutic potential to terminate chronic viral infections of the liver and possibly other persistent viral infections as well.

## Materials and Methods

### Ethics statement

All experiments involving mice were performed in the AAALAC accredited vivarium (Vertebrate Animal Assurance No. A3194-01) at The Scripps Research Institute. All animal studies follow the guidelines in the NIH Guide for the Care and Use of Laboratory Animals and are approved by The Scripps Research Institute Animal Care and Use Committee (Protocol # 08-0159).

### Mice

HBV transgenic mouse lineage 1.3.32 (inbred C57BL/6, H-2^b^) and lineage MC50 have been previously described [19], [37]. Lineage 1.3.32 expresses all of the HBV antigens and replicates HBV in the liver and kidney at high levels without any evidence of cytopathology. Lineage MUP-core 50 (MC50) (inbred C57BL/6, H-2^b^) expresses the HBV core protein in hepatocyte under the transcriptional control of the mouse major urinary protein (MUP) promoter. In all experiments, the mice were matched for age (8 weeks), sex (male), and (for the 1.3.32 animals) serum HBeAg levels in their serum before experimental manipulations. PD-1 deficient mice and CD11c.DOG mice (both inbred C57BL/6, H-2^b^), kindly provided by Drs. Arlene Sharpe and Günter Hämmerling, respectively, have been previously described [31], [45]. All experiments were approved by The Scripps Research Institute Animal Care and Use Committee.

### Peptide and recombinant vaccinia virus

Synthetic peptides corresponding to the previously described [27], [28], [71] HBV envelope (ENV)-specific CTL epitope, ENV28 (L^d^; IPQSLDSWWTSL) and HBV nucleocapsid protein (COR)-specific CTL epitope, COR93 (K^b^; MGLKFRQL) were purchased from Mimotope (Victoria, Australia). Recombinant vaccinia viruses that express the nucleocapsid protein (core) (subtype ayw) of HBV (designated cVac) and the major envelope protein (S) (subtype adw2) (HBs4) were kindly provided by H.J. Schlicht [30] and B. Moss [72], respectively.

### Generation of COR93-specific CD8^+^ T cell clone

A CD8^+^ CTL clone termed BC10, that is K^b^-restricted and specific for an epitope located between residues 93–100 in the HBV core protein (MGLKFRQL) (COR93), was generated in Balb/c (H-2^d^) by C57BL/6 (H-2^b^) F1 hybrids (CB6F1) that were immunized by standard DNA-prime/vaccinia boost immunization to induce an HBcAg specific CD8^+^ T cells response as previously described [21], [25]. Fourteen days after the booster immunization, mice were sacrificed and spleen cells were collected. 4×10^6^ spleen cells were cocultured with 1×10^5^ of irradiated RBL5 cell transfectants that express the HBV core protein (RBL5/c) in complete RPMI 1640 medium (GIBCO, Frederick, Md.) containing streptomycin (100 µg/ml), penicillin (100 U/ml), 2-mercaptoethanol (5×10^−5^ M), 10% fetal calf serum, and 2.5% EL-4 supernatant in 24-well plates (Costar, Cambridge, Mass.). The RBL5/c cell line was a gift from Dr. Jorg Reiman [71]. Seven days later, the spleen cells were restimulated with RBL5/c, and on day 14, they were cloned in 96-well round bottom plates (Costar) at 1 cell/well. After 2 to 3 weeks of repetitive stimulation, wells containing growing cells were expanded and four H-2^b^ restricted CTL clones were established. A CTL clone, termed BC10, was chosen for further characterization based on its superior cytolytic activity against RBL5/c. The fine antigen specificity of BC10 was confirmed by intracellular IFNγ production in response to a dominant, K^b^ restricted COR93-CTL epitope [71].

### Generation of T cell receptor (TCR) transgenic mice

TCR-α and -β cDNAs were synthesized from 20 ng of total messenger RNA extracted from COR93-specific CD8^+^ T cell clone BC10 and ENV28-specific CD8^+^ T cell clone 6C2 [27], [29], and amplified by PCR as described by Yoshida, et al [73]. The PCR products were cloned into the pGEM-T Easy Vector (Promega. Madison, WI) and then sequenced. The sequence analyses revealed that CTL clone BC10 expressed a TCR composed of Vα13.1JαNEW06 and Vβ8.1Jβ1.2, while the CTL clone 6C2 expressed a TCR composed of Vα4.1JαNEW and Vβ1.1Jβ2.5 chains. Flanking primers were designed to amplify the rearranged Vα13.1JαNEW06 and Vβ8.1Jβ1.2 genomic DNA based on genomic sequence from these TCR loci. PCR products were sequenced again and then inserted into the TCR expression cassettes pTα and pTβ [26], kindly provided by Dr. Diane Mathis. Prokaryotic DNA sequences were removed from both vectors and injected into fertilized CByB6F2 eggs as previously described [19]. Founders were screened by PCR and analyzed for the specific TCR expression on CD8^+^ T cells in the peripheral blood by FACS analysis. A founder, BC10.3, expressed TCR specific for COR93 epitope and was bred against C57BL/6 mice (H-2^b^) for 6 generation and then against CD45.1 mice (C57BL/6 background; H-2^b^) for at least 6 more generations. A founder, 6C2.36, expressed TCRs specific for ENV28 epitope and was bred against Balb/c mice (H-2^d^) for more than 6 generations before being mated with CD45.1 mice to produce H-2^bxd^ F1 hybrids.

### Adoptive transfer and vaccinia virus infection

Spleen cells were isolated from TCR transgenic mice BC10.3 (CD45.1^+^;H-2^b^) or 6C2.36xCD45.1 F1 hybrids (CD45.1^+^; H-2^bxd^) as previously described. Spleen cells from BC10.3 were transferred into either lineage 1.3.32 mice that were homozygous for HBV, or into MC50 mice heterozygous for the HBV core antigen, or into nontransgenic C57BL/6 mice (H-2^b^). In selected experiments, the mice were either intravenously infected with 2×10^7^ of recombinant vaccinia viruses expressing the HBV core antigen (cVac) or received saline as a control. Spleen cells from 6C2.36xCD45.1 F1 hybrids were transferred into HBV transgenic mice lineage 1.3.32× Balb/c F1 hybrids (H-2^bxd^) and syngeneic nontransgenic recipients. Groups of 3–4 mice were sacrificed at various time points after adoptive transfer and their livers, lymph nodes, and spleen were harvested for further analysis.

### Anti-CD40 and anti-CD62L antibody treatment

The FGK45 hybridoma producing rat IgG2a mAb against mouse CD40 (αCD40) was provided by Dr. A. Rolink (Basel Institute for Immunology, Basel, Switzerland) [43]. CD40 was purified from FGK45 culture supernatants as previously described [44]. Mice were intravenously injected with 100 µg of CD40 16 hours before adoptive transfer of HBV-specific naïve T cells. The mice were sacrificed at different time points after injection, and their livers were harvested for further analysis (see below). A monoclonal anti-CD62L antibody (clone Mel-14) was purchased from BD Bioscience, and mice were intraperitoneally administered with 100 µg of anti-CD62L mAb (αCD62L; clone Mel14) in 200 µl of PBS at 16, and 4 hours before adoptive transfer. If necessary, further doses of antibodies were administered on day 2 after adoptive transfer.

### Diphtheria Toxin (DTX) and Clodronate Liposome treatment

Diphtheria Toxin (DTX) was purchased from Sigma-Aldrich, dissolved in PBS, and intraperitoneally administered (200 ng/mouse) every other day. Clodronate Liposome and control Liposome were both purchased from Encapusula NanoSciences, and intravenously injected once (200 µl/mouse).

### Lymphmononuclear cell preparation

Spleen cells, lymph node cells, and intrahepatic lymphocytes (IHL) were prepared as previously described [21], [74]. Briefly, spleen cells and lymph node cells were isolated by pressing through a 70 µm cell strainer (Becton Dickinson) with the plunger of a 1-ml syringe and were washed three times with PBS and used for further analysis. For IHL isolation, livers were perfused with 10 ml of PBS via the portal vein to remove circulating lymphocytes and the liver cell suspension was pressed through a 70 µm cell strainer and digested with 10 ml of RPMI 1640 medium (Life Technologies), containing 0.02% (w/v) collagenase IV (Sigma) and 0.002% (w/v) DNase I (Sigma), for 40 minutes at 37°C. Cells were washed with RPMI 1640 and then overlaid on Percoll/Histopaque solution consisting of 12% Percoll (Pharmacia) and 88% Histopaque-1083 (Sigma-Aldrich). After centrifugation for 20 min at 1500× g, the IHLs were isolated at the interface. The lymphmononuclear cells were washed twice with RPMI 1640 medium and used for further analysis

### Isolation of primary hepatocytes, liver sinusoidal endothelial cells (LSECs), hepatic dendritic cells, and Kupffer cells

The livers of HBV transgenic mice lineage 1.3.32 were perfused slowly via the inferior vena cava with 25 ml of warm Liver Perfusion Medium (Gibco-Life Technologies) at a rate of 5 ml/minute, and then digested with 50–75 ml of warm Liver Digest Medium (Gibco-Life Technologies) at a rate of 5 ml/min. Following complete digestion of the liver (10–15 min.), the gall bladder was removed and the liver carefully excised. Cells were collected from the liver by disrupting the liver capsule and swirling the tissue in a petri dish containing Liver Digest Medium. Liver nonparenchymal cells (LNPCs) containing LSECs were separated from hepatocytes by centrifuging the cell suspension at 100× g for 2 min at room temperature. For LSEC isolation, the supernatant containing LNPCs was washed twice with RPMI 1640 (Cellgro), and the cell pellet was resupended with BD IMag Buffer at a concentration of 1×10^7^/ml. The cell suspension was incubated with biotinylated antibody specific for lymphatic vessel endotherial hyaluronan receptor 1 (LYVE-1) antibody (eBioscience) (20 µl for every 1×10^7^ of LNPCs) for 15 min on ice, washed twice, and resuspended in IMag buffer at a concentration of 2×10^7^/ml. The cell suspension was then incubated with BD IMag Streptavidin Particles Plus-DM (BD Bioscience) (50 µl for every 1×10^7^ of LNPCs) for 30 min on ice, and washed twice, and resuspended in IMag buffer at a concentration of 2 to 8×10^7^. LYVE-1^+^ cells were then positively selected using BD IMagnet (BD Bioscience) following the manufactures instruction, and then stained with PE-conjugated CD147, APC-conjugated CD31, and Alexa 700 conjugated CD45. The purity and viability of LSEC (CD147^+^CD31^+^CD45^−^) cells [75], [76] was routinely greater than 80% and 90%, respectively. For hepatocyte isolation, the pellet from the initial centrifugation step were washed in DMEM containing 10% FCS and centrifuged at 100× g for 2 min at room temperature. This washing step was repeated until the supernatant was no longer cloudy. Hepatocyte viability was routinely higher than 80%. For intrahepatic Dendritic cell (DC) and Kupffer cell (KC) isolation, intrahepatic lymphocytes (IHLs) were isolated as described in the previous section, and DCs and Kupffer cells were positively selected using biotinylated CD11c and CD11b and streptavidin Magnetic Particles (BD Biosciences), following the manufactures instruction. The purity of DCs and KCs were then analyzed by staining with PE-conjugated CD11b, APC-conjugated CD11c, PE-Cy7-conjugated F480, and FITC-conjugated Ly6G. The purities of DCs (CD11c^+^CD11b^+^Ly6G^−^) and KCs (CD11b^+^F480^+^CD11c^−^Ly6G^−^) were routinely greater than 50%, and their viabilities higher than 90%. All antibodies were purchased from BD Bioscience and eBioscience.

### Flowcytometric T cell analysis

Lymphmononuclear cells isolated from the liver, spleen, peripheral blood and lymph nodes were incubated with a mixture containing the COR93-dimer or ENV28-dimer, APC-, or Pacific Blue-conjugated anti-mouse CD8^+^, PE-Cy7 conjugated anti-mouse CD69, APC-conjugated anti-mouse CD25 or CD62L, FITC-conjugated anti-mouse CD45.1, and PE-conjugated PD-1 or CTLA-4 (BD Bioscience) for 1 hour on ice. After washing, the cells were incubated for 30 minutes with APC-conjugated anti-mouse IgG at 4°C to detect dimer positive cells. Dimer without peptide was used as a control. Intracellular cytokine staining (ICS) was performed using PE- or APC-conjugated anti-mouse IFNγ, APC-conjugated anti-mouse Granzyme B (GrB) (Caltag) after incubating for 5 hours at 37°C in the presence of brefeldin A (BFA), as previously described [21], [25], [49]. All antibodies were purchased from BD Bioscience and eBioscience.

### Tissue DNA and RNA analyses

Total liver DNA and RNA were analyzed for HBV replicative intermediates by Southern blot, and for HBV RNA by Northern blot, exactly as previously described [19], [20]. The relative abundance of specific DNA and RNA molecules was determined by phosphor imaging analysis, using the Optiquant image analysis software (Packard).

### Biochemical analyses

The extent of hepatocellular injury was monitored by measuring sALT activity at multiple time points after treatment as previously described [20].

### Statistical analysis

Student t test was performed using Microsoft Excel. Data are depicted as the mean ± SD, and P values<0.05 were considered significant: *P<0.05, **P<0.01, ***P<0.001.
