# Correction: CD40 Activation Rescues Antiviral CD8^+^ T Cells from PD-1-Mediated Exhaustion

**DOI:** 10.1371/journal.ppat.1006086

**Published:** 2016-12-07

**Authors:** Masanori Isogawa, Josan Chung, Yasuhiro Murata, Kazuhiro Kakimi, Francis V. Chisari

In the Results section and the Materials and Methods section, the alpha chain of the ENV28-specific T cell receptor (TCR) is incorrectly identified as Vα4.1JαNEW. The correct alpha chain usage of that TCR is Vα4.11JαNEW.

## References

[1] Isogawa M, Chung J, Murata Y, Kakimi K, Chisari FV (2013) CD40 Activation Rescues Antiviral CD8^+^ T Cells from PD-1-Mediated Exhaustion. PLoS Pathog 9(7): e1003490 doi: 10.1371/journal.ppat.1003490 23853599 10.1371/journal.ppat.1003490 PMC3708877

