# Correction: CD40 Activation Rescues Antiviral CD8^+^ T Cells from PD-1-Mediated Exhaustion

**DOI:** 10.1371/journal.ppat.1006416

**Published:** 2017-05-31

**Authors:** Masanori Isogawa, Josan Chung, Yasuhiro Murata, Kazuhiro Kakimi, Francis V. Chisari

In the Results section and the Materials and Methods section, the beta chain of the COR93-specific T cell receptor (TCR) is incorrectly identified as Vβ8.1Jβ1.2. The correct beta chain usage of that TCR is Vβ8.3Jβ1.2.
